# An Exergy-Enhanced Improved IGDT-Based Optimal Scheduling Model for Electricity–Hydrogen Urban Integrated Energy Systems

**DOI:** 10.3390/e27070748

**Published:** 2025-07-13

**Authors:** Min Xie, Lei Qing, Jia-Nan Ye, Yan-Xuan Lu

**Affiliations:** School of Electric Power, South China University of Technology, Guangzhou 510641, China; 202321015040@mail.scut.edu.cn (L.Q.); 202021015844@mail.scut.deu.cn (J.-N.Y.); 202220114135@mail.scut.edu.cn (Y.-X.L.)

**Keywords:** exergy efficiency, stochastic optimization, SOI-IGDT, multiple uncertainties, integrated energy systems

## Abstract

Urban integrated energy systems (UIESs) play a critical role in facilitating low-carbon and high-efficiency energy transitions. However, existing scheduling strategies predominantly focus on energy quantity and cost, often neglecting the heterogeneity of energy quality across electricity, heat, gas, and hydrogen. This paper presents an exergy-enhanced stochastic optimization framework for the optimal scheduling of electricity–hydrogen urban integrated energy systems (EHUIESs) under multiple uncertainties. By incorporating exergy efficiency evaluation into a Stochastic Optimization–Improved Information Gap Decision Theory (SOI-IGDT) framework, the model dynamically balances economic cost with thermodynamic performance. A penalty-based iterative mechanism is introduced to track exergy deviations and guide the system toward higher energy quality. The proposed approach accounts for uncertainties in renewable output, load variation, and Hydrogen-enriched compressed natural gas (HCNG) combustion. Case studies based on a 186-bus UIES coupled with a 20-node HCNG network show that the method improves exergy efficiency by up to 2.18% while maintaining cost robustness across varying confidence levels. These results underscore the significance of integrating exergy into real-time robust optimization for resilient and high-quality energy scheduling.

## 1. Introduction

Integrated energy systems (IESs) are rapidly evolving to meet the increasing demands of urban decarbonization, the electrification of end-use sectors, and the growing need for multi-energy coordination and interconnection [1]. By coupling various energy carriers such as electricity, heating, cooling, natural gas, and hydrogen, these systems offer a promising pathway to enhance overall energy efficiency and system flexibility [2].

However, conventional IES scheduling models are primarily designed to optimize energy “quantity” or minimize operational costs [3,4]. While effective from an economic standpoint, these models largely overlook the inherent differences in thermodynamic quality among energy carriers—such as the high-grade nature of electricity compared to low-grade thermal energy, or the variable quality of hydrogen-rich gases [5,6]. Treating diverse forms of energy as equivalent in optimization models, despite their distinct thermodynamic potentials, may lead to misleading conclusions. Relying solely on quantity-based performance indicators is insufficient to capture the true effectiveness and rationality of energy utilization in such complex systems [7,8].

To address this gap, the concept of exergy has been increasingly adopted in system analysis. Exergy is defined as the maximum useful work obtainable from a given form of energy when brought into equilibrium with its environment. It provides a unified and physically grounded metric for assessing the quality and usability of energy across different carriers [9]. By quantifying the inherent irreversibilities in energy conversion, transport, and storage processes, exergy-based models can reveal hidden inefficiencies that are often obscured in energy-only assessments [10,11,12]. This enables deeper insights into where energy quality losses occur and how they can be minimized [13,14]. Despite its analytical value, exergy has mostly been used retrospectively as an evaluation tool in existing studies, rather than as a forward-looking input for real-time operational decision-making [15,16]. This separation between evaluation and control limits the ability of IES to adaptively optimize operations for thermodynamic performance, especially under dynamic and uncertain conditions [17,18,19].

In practice, the operation of IES is subject to various uncertainties, including the stochastic fluctuations of renewable generation (e.g., wind and solar), time-varying energy demand, and the complex and unstable combustion characteristics of hydrogen-rich natural gas in gas turbines. These uncertainties pose significant challenges for effective real-time scheduling [20,21]. Moreover, several recent studies have explored uncertainty-aware scheduling in integrated energy systems. For example, Zhang et al. developed a fuzzy-stochastic dispatch model to manage the uncertainty of wind power and load under a carbon trading mechanism [22]. Another study proposed a robust chance-constrained model to optimize supply-demand scheduling in power markets, ensuring reserve requirements under confidence-level constraints [23]. More recently, a hybrid stochastic and chance-constrained bidding strategy was introduced [24], incorporating distributed renewable generation and multi-energy load uncertainties into a day-ahead electricity market framework. While these methods enhance robustness under uncertain conditions, they primarily focus on economic objectives and market coordination. Few of them consider the coupling effects of multiple uncertainty sources, such as renewables and fuel characteristics, and none of them have integrated thermodynamic performance indicators such as exergy efficiency into the scheduling framework. This underscores the need for more adaptive and exergy-aware optimization frameworks under compound uncertainty environments.

While the Information Gap Decision Theory (IGDT) has emerged as a powerful tool for decision-making under severe uncertainty, offering a structured risk-averse optimization framework, traditional implementations have focused exclusively on the degradation of economic objectives [25]. They fail to account for exergy-related performance metrics, which are essential for ensuring high-quality energy utilization in IES [26]. In view of the current research status, IGDT still has some limitations. First, the description of uncertain variables is often oversimplified and fails to capture their inherent randomness. Second, the deviation factor must be manually preset, which introduces subjectivity into the modeling process. Third, conventional IGDT models primarily focus on economic robustness while neglecting thermodynamic characteristics, making them less effective in exergy-sensitive systems such as EHUIES. Therefore, there is a need to improve the IGDT framework by integrating thermodynamic performance indicators and more accurate representations of uncertainty.

To overcome these challenges, this paper proposes a novel optimization framework that explicitly incorporates exergy efficiency into the scheduling process. Termed Stochastic Optimization–Improved IGDT (SOI-IGDT), the framework integrates exergy indicators into a dynamic, uncertainty-aware optimization model. Specifically, an enhanced IGDT method with exergy-oriented features is developed for constructing an optimal scheduling model for electricity–hydrogen urban integrated energy systems (EHUIESs) under multiple uncertainties. In this model, cost robustness and exergy efficiency tracking are jointly managed through a deviation penalty mechanism, which is iteratively updated based on the system’s fluctuating states. This ensures that the system not only withstands external uncertainties but also maintains high thermodynamic performance across the entire scheduling horizon.

A case study based on a real-world urban electricity–hydrogen–natural gas integrated energy system is conducted to validate the proposed SOI-IGDT approach. Simulation results demonstrate that the model significantly improves energy efficiency while maintaining operational resilience across multiple confidence levels, highlighting its suitability for deployment in intelligent and sustainable urban energy infrastructures.

## 2. Materials and Methods

### 2.1. Structure of Electric-Hybrid Hydrogen-Gas Urban Integrated Energy System

**Figure 1:**
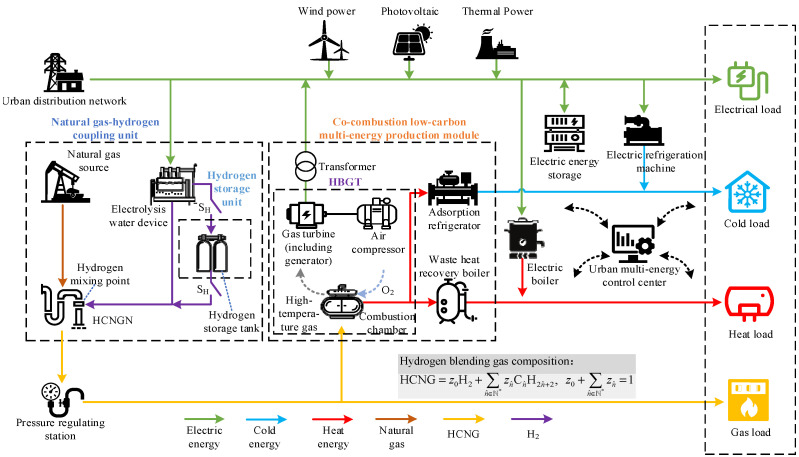
Structure of an EHUIES.

In order to reasonably construct the EHUIES low-carbon optimization scheduling model, this section introduces the EHUIES structure in detail, among which the natural gas–hydrogen coupling unit and the co-firing low-carbon multi-energy production module are the core parts of an EHUIES, as shown in Figure 1.

As illustrated in Figure 1, the urban distribution network and the hydrogen-enriched compressed natural gas network (HCNGN) serve as key hubs for hydrogen production and blending within the EHUIES. In the natural gas–hydrogen coupling unit, the water electrolysis system enables the conversion of electrical energy into hydrogen. The produced hydrogen is then blended with natural gas from the gas supply source via the HCNGN. A hydrogen storage tank, located in the hydrogen storage unit, acts as a critical energy buffer, allowing the system to decouple hydrogen production from usage. The hydrogen blending process is regulated by the on/off status of the control switch SH.

The co-combustion low-carbon multi-energy generation module comprises transformers, hydrogen-blended gas turbines (HBGTs), adsorption chillers, and waste heat recovery boilers. Each HBGT consists of an air compressor, combustion chamber, and gas turbine (including its generator section). The HCNG fuel is supplied via a pressure regulating station, which ensures that the gas mixture meets the required compositional specifications. Waste heat from the HBGTs is recovered and utilized by the adsorption chillers and WHRBs, enhancing system energy efficiency.

The energy supply section of EHUIES, connected to the urban distribution network, integrates wind power, photovoltaic (PV) power, and thermal power sources. The energy conversion units include electric boilers and electric chillers, while battery energy storage systems serve as the primary means of electric energy storage. On the demand side, EHUIES supports four types of loads: electricity, heating, cooling, and gas.

### 2.2. Low-Carbon Optimization Dispatch Model for Electricity–Hydrogen–Natural Gas Hybrid Urban Integrated Energy System

This section introduces the low-carbon optimal scheduling model of EHUIES, as depicted in Figure 1, focusing on the formulation of the objective function and the associated system constraints.

#### 2.2.1. Optimization Goals

The low-carbon optimal scheduling model of the EHUIES aims to minimize the total system operating cost *F*, which serves as the objective function. The total cost *F* consists of multiple components, including the operation cost of thermal power units *F*_pg_, the operation cost of water electrolysis units *F_we_*, wind power curtailment cost *F*_aw_, carbon emission cost *F*_ce_, natural gas supply cost from the external source *F*_ng_, HCNG purchase cost of the user unit *F*_hg_, electricity purchase cost of the user unit *F*_pe_, operation and maintenance cost of system equipment *F*_om_, and the cost associated with the hydrogen storage unit *F*_hs_. The variable *μ*_hs_ denotes the operating state of the hydrogen storage unit, which is primarily determined by the on/off status of the control switch S_H_. This variable plays a key role in subsequent scenario analyses. The objective function is expressed as follows:

(1) $$\min\;F=F_{\mathrm{pg}}+F_{\mathrm{we}}+F_{\mathrm{aw}}+F_{\mathrm{ce}}+F_{\mathrm{ng}}+F_{\mathrm{hg}}+F_{\mathrm{pe}}+F_{\mathrm{om}}+\mu_{\mathrm{hs}}F_{\mathrm{hs}},\mu_{\mathrm{hs}}=\left\{\begin{array}{l} 0,{\;S}_{H}\;\mathrm{is}\;\mathrm{open}\\ 1,{\;S}_{H}\;\mathrm{is}\;\mathrm{closed} \end{array}\right.$$

The operation cost of thermal power generation units, denoted as *F*_pg_, can be calculated as follows:

(2) $$F_{\mathrm{pg}}=\sum_{t=1}^{T}\sum_{g=1}^{G}\left(a_{g}P_{g,t}^{2}+b_{g}P_{g,t}+c_{g}\right)$$

where *T* is the scheduling time horizon, *G* is the total number of thermal power units, *a_g_*, *b_g_*, *c_g_* are the cost coefficients of thermal power unit *g*, and *P_g_*_,*t*_ is the power output of unit *g* at time *t*.

The operation cost of the water electrolysis units, denoted as *F*_we_, is expressed as follows:

(3) $$F_{\mathrm{we}}=\zeta_{\mathrm{we}}\sum_{t=1}^{T}\sum_{n=1}^{N}P_{\mathrm{we},n,t}$$

where *N* is the number of water electrolysis units, *ζ*_we_ is the operation cost coefficient of the water electrolysis unit, *P*_we,*n*,*t*_ is the power consumption of electrolysis unit *n* at time *t*.

During the wind power integration process, the associated wind curtailment cost, denoted as *F*_aw_, is calculated as follows:

(4) $$F_{\mathrm{aw}}=\delta_{\mathrm{aw}}\sum_{t=1}^{T}\sum_{w=1}^{W}\left({\tilde{P}}_{w,t}-P_{w,t}\right)$$

where *N* is the number of wind farms, *δ_aw_* is the wind curtailment price, ${\tilde{P}}_{w,t}$ is the forecasted power of wind farm *w* at time *t*, *P_w_*_,_ is the actual utilized power of wind farm *w* at time *t.*

The carbon emission cost, denoted as *F*_ce_, is calculated as follows:

(5) $$F_{\mathrm{ce}}=\xi_{\mathrm{ce}}\sum_{t=1}^{T}\sum_{m=1}^{M}Q_{\mathrm{cab},m,t}$$

where *M* is the number of HBGTs; *ξ_ce_* is the carbon tax price.

Since the natural gas source supplies natural gas to the HCNGN, which is then blended with the injected hydrogen in a non-fixed ratio before being sold as HCNG to user units, the natural gas supply cost *F*_ng_ and the user-side HCNG purchase cost *F*_hg_ are calculated as follows:

(6) $$F_{\mathrm{ng}}=\gamma_{\mathrm{ng}}\sum_{t=1}^{T}\sum_{s=1}^{S}q_{s,t}\Delta t$$

(7) $$F_{\mathrm{hg}}=\sum_{t=1}^{T}\sum_{m=1}^{M}\gamma_{\mathrm{hg},t}V_{\mathrm{hg},m,t}$$

where *M* is the number of natural gas sources, *γ_ng_* is the natural gas price, *γ*_*hg*,*t*_ is the price of HCNG at time *t*, Δ*t* is the scheduling time interval, and *q*_*s*,*t*_ is the natural gas supply flow rate from source *s* at time *t*.

The electricity purchase cost of the user unit, denoted as *F*_pe_, is calculated as follows:

(8) $$F_{\mathrm{pe}}=\sum_{t=1}^{T}\sum_{l=1}^{L}\gamma_{\mathrm{pe},t}P_{\mathrm{pe},l,t}$$

where *L* is the number of urban terminal user units, *γ*_pe,*t*_ is the price of electricity sold by the urban distribution network to user units at time *t*, and *P*_*pe*,*l*,*t*_ is the power purchased by the *l*-th user unit at time *t*.

The operation and maintenance cost *F*_om_ consists of the costs associated with the operation and maintenance of electrical energy storage systems, HBGTs, electric chillers, adsorption chillers, electric boilers, and waste heat recovery boilers. It is expressed as follows:

(9) $$F_{\mathrm{om}}=\sum_{t=1}^{T}\left[\varphi_{\mathrm{eh}}P_{\mathrm{eh},t}+\varphi_{\mathrm{ht}}\sum_{m=1}^{M}P_{\mathrm{ht},m,t}+\varphi_{\mathrm{ec}}P_{\mathrm{ec},t}+\varphi_{\mathrm{rh}}H_{\mathrm{rh},\mathrm{in},t}+\right.\left.\varphi_{\mathrm{ac}}H_{\mathrm{ac},\mathrm{in},t}+\varphi_{\mathrm{bat}}\sum_{u=1}^{U}\left(P_{\mathrm{ch},u,t}+P_{\mathrm{dis},u,t}\right)\right]$$

where *U* is the number of electrical energy storage units, *φ_eh_*, *φ*_ht_, *φ*_ec_, *φ*_rh_, *φ*_ac_, and *φ*_bat_ represent the operation and maintenance cost coefficients of the electric boiler, electric chiller, waste heat recovery boiler, adsorption chiller, HBGT, and electrical energy storage unit, respectively; *P*_eh,*t*_ is the electric power consumed by the electric boiler at time *t*, *P*_ec,*t*_ is the electric power consumed by the electric chiller at time *t*, and *P*_ch,*u*,*t*_ and *P*_dis,*u*,*t*_ are the charging and discharging power of the *u*-th electrical energy storage unit at time *t*, respectively. *H*_rh,in,*t*_ is the thermal input power to the waste heat recovery boiler at time *t*, and *H*_ac,in,*t*_ is the thermal input power to the adsorption chiller at time *t.*

The cost of the hydrogen storage unit *F*_hs_ primarily consists of the hydrogen storage investment cost and the hydrogen storage operational cost, which can be expressed as follows:

(10) $$F_{\mathrm{hs}}=\underset{investment\;cost}{\underset{︸}{c_{\mathrm{invest},\mathrm{hst}}N_{\mathrm{hst}}}}+\underset{operational\;cost}{\underset{︸}{\alpha_{\mathrm{hs}}\sum_{t=1}^{T}\sum_{h=1}^{N_{h}}(q_{\mathrm{hs},h,t}^{\mathrm{in}}+q_{\mathrm{hs},h,t}^{\mathrm{out}})\Delta t}}$$

where *N_h_* is the number of hydrogen storage units, *c*_*invest*,*hst*_ is the price of a hydrogen storage tank, *N*_hst_ is the number of hydrogen storage tanks, *α*_hs_ is the hydrogen storage cost coefficient, $q_{\mathrm{hs},h,t}^{\mathrm{in}}$ and $q_{\mathrm{hs},h,t}^{\mathrm{out}}$ are the hydrogen inflow and outflow rates of the *h*-th hydrogen storage unit at time *t*, respectively.

#### 2.2.2. Constraints

##### Operating Constraints of Urban Distribution Systems

(1) Thermal Power Unit Operation Constraints

The operation of thermal power units is subject to power output constraints, which are specifically expressed as follows:

(11) $$P_{g,\min}\leq P_{g,t}\leq P_{g,\max}$$

The ramp rate constraints for the operation of thermal power units can be expressed as follows:

(12) $$\left\{\begin{array}{l} P_{g,t-1}-P_{g,t}\leq {\hat{r}}_{d,g}\Delta t\\ P_{g,t}-P_{g,t-1}\leq {\hat{r}}_{u,g}\Delta t \end{array}\right.$$

where ${\hat{r}}_{d,g}$ and ${\hat{r}}_{u,g}$ are the downward-climbing rate and upward-climbing rate of the *g*-th thermal power unit, respectively.

(2) Operational Constraints of Wind Farms

In the deterministic day-ahead optimal scheduling of EHUIES, it is assumed that the actual power output of the wind farm does not exceed its forecasted power, which can be expressed as follows:

(13) $$0\leq P_{w,t}\leq {\tilde{P}}_{w,t}$$

(3) Power Balance Constraint

Taking the urban distribution network as the core framework for electrical energy, the operation of the water electrolysis units, thermal power units, wind farms, and photovoltaic power stations are comprehensively considered, along with the electricity purchasing behavior of some user units and the electrical load of the urban distribution network. The power balance of the urban distribution network is calculated according to the following equation:

(14) $$\sum_{g=1}^{G}P_{g,t}+\sum_{w=1}^{W}P_{w,t}+P_{\mathrm{vps},t}^{\mathrm{UD}}=\sum_{l=1}^{L}P_{\mathrm{ep},l,t}+\sum_{n=1}^{N}P_{\mathrm{we},n,t}+P_{\mathrm{load},t}^{\mathrm{UD}}$$

where $P_{\mathrm{vps},t}^{\mathrm{UD}}$ is the predicted power generation of photovoltaic power stations in urban distribution network at time *t*, $P_{\mathrm{load},t}^{\mathrm{UD}}$ is electric load of urban distribution network at time *t*, which is given by $P_{\mathrm{load},t}^{\mathrm{UD}}=\sum_{d=1}^{N_{d}}P_{\mathrm{load},d,t}$, where *N*_d_ is the number of load nodes, and *P*_*load*,*d*,*t*_ is the electrical load at load node *d* in the urban distribution network at time *t*.

(4) Transmission Capacity Constraints of Urban Distribution Network Lines

For the power flow constraints in the urban distribution network, the transmission capacity limits of each power line must be considered to ensure compliance with the actual operating conditions of the urban distribution network. These constraints can be expressed as follows:

(15) $$\begin{array}{l} -P_{x,\max}^{\mathrm{tc}}\leq \sum_{g=1}^{G}l_{x,g}P_{g,t}+\sum_{w=1}^{W}l_{x,w}P_{w,t}+l_{x,\mathrm{vps}}P_{\mathrm{vps},t}^{\mathrm{UD}}-\sum_{d=1}^{N_{d}}l_{x,d}P_{\mathrm{load},d,t}-\\ \sum_{l=1}^{L}l_{x,l}P_{\mathrm{ep},l,t}-\sum_{n=1}^{N}l_{x,n}P_{\mathrm{we},n,t}\leq P_{x,\max}^{\mathrm{tc}} \end{array}$$

where $P_{x,\max}^{\mathrm{tc}}$ is the upper limit of transmission capacity of line *x*, $l_{x,g}$, $l_{x,w}$, $l_{x,\mathrm{vps}}$, $l_{x,d}$, $l_{x,l}$ and $l_{x,n}$ are the transfer distribution factors of the *g*-th thermal power unit, the *w*-th wind farm, the photovoltaic power station, the load node *d*, the *l*-th user unit, and the *n*-th water electrolysis device on the line *x*.

##### Operating Constraints of the Hydrogen-Enriched Natural Gas Network

Assuming that HCNG flows in the HCNGN under constant temperature and variable pressure, the inlet temperature of the HBGT is derived from the node outlet pressure (after secondary regulation) using the ideal gas law. Considering HCNG component tracking, the HCNGN must satisfy the following constraints:

(1) Pipeline Flow–Pressure Relationship Constraint

Assuming HCNG flows directionally, the pressure–flow relationship in the gas network is formulated as follows:

(16) $$q_{ij,t}\left|q_{ij,t}\right|=\frac{\pi^{2}R}{64}\cdot {\left(\frac{T_{\mathrm{st}}}{p_{\mathrm{st}}}\right)}^{2}\cdot \frac{p_{i,t}^{2}-p_{j,t}^{2}}{\rho_{i,t}L_{ij}T_{\mathrm{hg}}\Phi_{ij}f_{ij}}\cdot D_{ij}^{5}$$

where *q*_*ij*,*t*_ is the pipeline flow between gas network nodes *i* and *j* at time *t*, *p_i_*_,*t*_ is the pressure of gas network node *i* at time *t*, *T*_st_ is HCNG temperature under standard conditions, *p*_st_ is HCNG pressure under standard conditions, *D_ij_*, *L_ij_*, and *f_ij_* are the pipe diameter, pipe length, and friction coefficient between gas network nodes *i* and *j*, respectively. *T_hg_* is HCNG pipeline temperature, *Φ_ij_* is the compression factor between gas grid nodes *i* and *j*.

Within safety limits, the HCNGN must satisfy both pipeline flow and node pressure constraints, as shown below.

(17) $$-q_{ij,\max}\leq q_{ij,t}\leq q_{ij,\max}$$

(18) $$p_{i,\min}\leq p_{i,t}\leq p_{i,\max}$$

(2) Energy Balance Constraint at Gas Network Nodes

**Figure 2:**
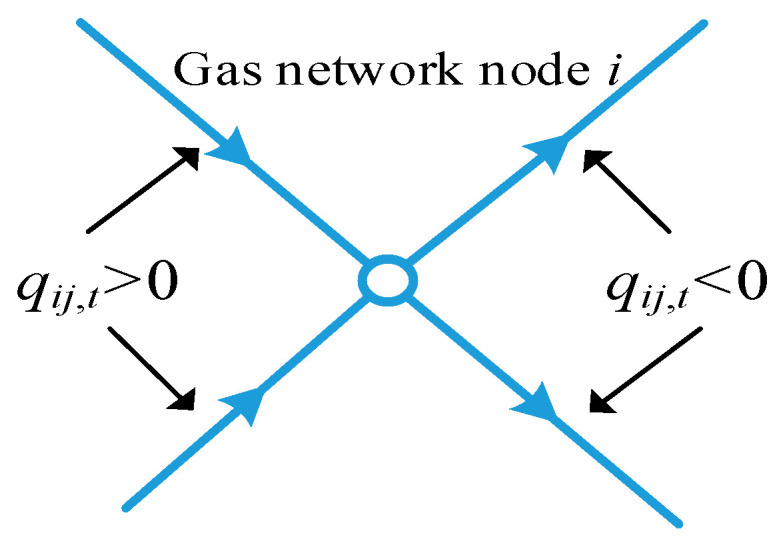
Positive direction of pipeline flow.

In the HCNGN, the pipeline flow into node *i* is defined as positive, as shown in Figure 2.

Any gas network node balances energy flow (power) and is related to the gas source points, hydrogen blending points, and other adjacent gas network nodes. Additionally, the reference flow direction in the HCNGN pipelines needs to be defined, where *Lq*(*j*,*i*) represents the set of all pipelines with reference direction from node *j* to node *i*, and *Lq*(*i*,*j*) represents the set of all pipelines with reference direction from node *i* to node *j*. Therefore, the energy balance at a gas network node in the HCNGN can be calculated using the following equation:

(19) $$\begin{array}{l} \chi_{H_{2},i}q_{H_{2},i,t}{\hat{H}}_{\mathrm{gas},z}+\chi_{i,s}q_{s,t}{\hat{H}}_{\mathrm{ng},s}+\sum_{ij\in L_{q}(j,i)}u_{ij,t}q_{ij,t}{\hat{G}}_{\mathrm{hg},j,t}-\sum_{ij\in L_{q}(i,j)}u_{ij,t}q_{ij,t}{\hat{G}}_{\mathrm{hg},i,t}+\\ \;\sum_{ij\in L_{q}(i,j)}\delta_{ij,t}q_{ij,t}{\hat{G}}_{\mathrm{hg},j,t}-\sum_{ij\in L_{q}(j,i)}\delta_{ij,t}q_{ij,t}{\hat{G}}_{\mathrm{hg},i,t}=E_{d,i,t}+E_{\mathrm{ht},i,t},\;z\in {\hat{z}}_{H_{2}} \end{array}$$

where $\chi_{H_{2},i}$ is the hydrogen mixing status of gas network node *i*, which can be 0 or 1, ${\hat{H}}_{\mathrm{gas},z}$ is high calorific value of gas component *z*, ${\hat{H}}_{\mathrm{ng},s}$ is the high calorific value of natural gas from the *s*-th gas source, ${\hat{G}}_{\mathrm{hg},i,t}$ is the HCNG calorific value of gas grid node *i* at time *t*, *E*_*d*,*i*,*t*_ and *E*_*ht*,*i*,*t*_ are the conventional gas load and HBGT load of gas grid node i at time t, respectively. *u_ij_*_,*t*_ and *δ_ij_*_,*t*_ are the directional indicator variables for the actual pipeline flow between gas network nodes *i* and *j* at time *t*, representing the positive and negative flow directions, respectively. Their calculation formulas are as follows:

(20) $$\left\{\begin{array}{l} \varepsilon_{ij,t}=\mathrm{sgn}(q_{ij,t})\\ u_{ij,t}=\frac{\left|\varepsilon_{ij,t}\right|+\varepsilon_{ij,t}}{2}\\ \delta_{ij,t}=\frac{\left|\varepsilon_{ij,t}\right|-\varepsilon_{ij,t}}{2} \end{array}\right.$$

where *sgn*(·) is the sign function, *ε*_*ij*,*t*_ is the actual pipeline flow direction between gas network nodes *i* and *j* at time *t*.

The supply flow constraint of natural gas sources is expressed as follows:

(21) $$q_{s,\min}\leq q_{s,t}\leq q_{s,\max}$$

(3) Pipeline Average Pressure Constraint

Based on the Soave–Redlich–Kwong equation of state, its form is further improved to describe the average pipeline pressure, which is expressed as follows:

(22) $$\left\{\begin{array}{c} p_{\mathrm{av},ij,t}=\frac{2}{3}\cdot \left(p_{i,t}+p_{j,t}-\frac{p_{i,t}p_{j,t}}{p_{i,t}+p_{j,t}}\right)\\ \Phi_{ij}^{3}-\Phi_{ij}^{2}+\Phi_{ij}\left(A_{ij,t}-B_{ij,t}-B_{ij,t}^{2}\right)-A_{ij,t}B_{ij,t}=0\\ A_{ij,t}=0.42747\cdot \frac{p_{\mathrm{av},ij,t}}{T_{\mathrm{hg}}^{2}}\cdot {\left(\sum_{z\in Z}\frac{N_{z,i,t}T_{c,z}\phi_{z}}{\sqrt{p_{c,z}}}\right)}^{2}\\ B_{ij,t}=0.08664\cdot \frac{p_{\mathrm{av},ij,t}}{T_{\mathrm{hg}}}\cdot \sum_{z\in Z}\frac{N_{z,i,t}T_{c,z}}{p_{c,z}}\\ \phi_{z}=1+\left(0.48+1.574\omega_{z}-0.176\omega_{z}^{2}\right)\cdot \left(1-\sqrt{\frac{T_{\mathrm{hg}}}{T_{c,z}}}\right) \end{array}\right.$$

where *p*_av,*ij*,*t*_ is average pipeline pressure between gas grid nodes *i* and *j* at time *t*, *T*_c,*z*_ and *p*_c,*z*_ are critical temperature and critical pressure of gas component *z*, *Z_ij_* is the pipeline compression coefficient between gas network nodes *i* and *j*, *A_ij_*_,*t*_ and *B_ij_*_,*t*_ are the HCNG state parameters between gas grid nodes *i* and *j* at time *t*, *ϕ_z_* is the eccentricity coefficient conversion parameter of gas component *z*.

(4) Calculation of Gas Component Molar Fractions

During the HCNGN balancing process, the HCNG composition varies at each gas network node. Based on the node flow balance, the molar fraction of gas component *z* at node *i* and time *t*, *N_z_*_,*i*,*t*_ can be calculated using the following equation:

(23) $$\begin{array}{l} N_{z,i,t}=\left(\chi_{H_{2},i}N_{H_{2},z,i,t}q_{H_{2},i,t}+\chi_{i,s}N_{\mathrm{ng},z,i,t}q_{s,t}+\sum_{ij\in L_{q}(j,i)}u_{ij,t}q_{ij,t}N_{z,j,t}-\sum_{ij\in L_{q}(i,j)}u_{ij,t}q_{ij,t}N_{z,i,t}+\right.\\ \sum_{ij\in L_{q}(i,j)}\delta_{ij,t}q_{ij,t}N_{z,j,t}-\sum_{ij\in L_{q}(j,i)}\delta_{ij,t}q_{ij,t}N_{z,i,t}/\left(\chi_{H_{2},i}q_{H_{2},i,t}+\chi_{i,s}q_{s,t}+\sum_{ij\in L_{q}(j,i)}q_{ij,t}-\sum_{ij\in L_{q}(i,j)}q_{ij,t}\right) \end{array}$$

where $N_{H_{2},z,i,t}$ is the molar fraction of gas component *z* in the hydrogen added to the gas grid node *i* at time *t*, $N_{\mathrm{ng},z,i,t}$ is the molar fraction of gas component *z* in the natural gas supplied by the gas source of gas grid node *i* at time *t*.

(5) Compressor and Pressure Regulating Station Operation Constraints

Compressors and pressure regulating stations assist HCNGN in maintaining safe and stable HCNG flow by performing secondary pressure regulation at gas network node outlets, ensuring suitable initial combustion conditions for HBGT. Their operational constraints are given by the following equations:

(24) $$\left\{\begin{array}{l} p_{j,t}=r_{ij,t}p_{i,t}\\ T_{\mathrm{in},i,t}=\frac{\varpi_{\mathrm{tf}}p_{i,t}}{Rk_{i,t}} \end{array}\right.$$

where *r_ij_*_,*t*_ is the compression ratio between gas network nodes *i* and *j* at time *t*, *ϖ_tf_* is the pressure conversion factor, and *k_i_*_,*t*_ is the pressure regulation ratio of the outlet side of the gas network node *i* at time *t*.

##### Operational Constraints of Urban End-User Units

To accurately represent EHUIES operation, equipment classification and power balance are detailed for different urban end-user types based on Figure 1. User units are categorized into residential communities, university campuses, and industrial parks. Residential and university areas mainly include HBGT, photovoltaic systems, and electrical energy storage, focusing on electrical power balance. Industrial parks incorporate HBGT, PV units, electric boilers, chillers, waste heat recovery boilers, adsorption chillers, and energy storage, requiring electrical, thermal, and cooling power balance.

(1) Charging and Discharging Constraints of Electrical Energy Storage

(25) $$E_{\mathrm{st},u,t}=E_{\mathrm{st},u,t-1}+\left(\eta_{\mathrm{st}}P_{\mathrm{ch},u,t}-\frac{P_{\mathrm{dis},u,t}}{\eta_{\mathrm{st}}}\right)\Delta t$$

(26) $$0\leq E_{\mathrm{st},u,t}\leq E_{\mathrm{st},u,\max}$$

where *E*_st,*u*,*t*_ is the capacity of the *u*-th energy storage at time *t*, and *η*_st_ is the conversion efficiency of the energy storage.

The state transition between charging and discharging of electrical energy storage systems is subject to a limited number of cycles. Moreover, charging and discharging cannot occur simultaneously, and the respective charging and discharging power must not exceed their allowable maximum values. The constraints can be expressed as follows:

(27) $$\left\{\begin{array}{c} \begin{array}{c} \sum_{t=1}^{T}\left|U_{\mathrm{ch},u,t}-U_{\mathrm{dis},u,t}\right|\leq N_{\mathrm{bat},u}\\ U_{\mathrm{dis},u,t}+U_{\mathrm{ch},u,t}\leq 1 \end{array}\\ \begin{array}{l} 0\leq P_{\mathrm{ch},u,t}\leq U_{\mathrm{ch},u,t}P_{\mathrm{ch},u,\max}\\ 0\leq P_{\mathrm{dis},u,t}\leq U_{\mathrm{dis},u,t}P_{\mathrm{dis},u,\max} \end{array} \end{array}\right.$$

where *U*_ch,*u*,*t*_ and *U*_dis,*u*,*t*_ are the charging and discharging states of the *u*-th energy storage at time *t*, respectively, *P*_ch,*u*,max_ and *P*_dis,*u*,max_ are the maximum charging and discharging powers allowed by the *u*-th energy storage, respectively, and *N*_bat,*u*_ is the number of charging and discharging conversions of the *u*-th energy storage.

(2) Operational Constraints of Waste Heat Recovery Boilers and Adsorption Chillers

The input heat for both the waste heat recovery boiler and the adsorption chiller is supplied by the HBGT combustion chamber. Their collected heat power must not exceed their respective maximum allowable limits. The operational constraints are given as follows:

(28) $$\left\{\begin{array}{c} \begin{array}{l} H_{\mathrm{rh},t}=\eta_{\mathrm{rb},h}H_{\mathrm{rh},\mathrm{in},t}\beta_{\mathrm{rb},h}\\ C_{\mathrm{ac},t}=\eta_{\mathrm{ar},c}H_{\mathrm{ac},\mathrm{in},t}\beta_{\mathrm{ar},c} \end{array}\\ \begin{array}{l} 0\leq H_{\mathrm{rh},\mathrm{in},t}\leq H_{\mathrm{rh},\mathrm{in},\max}\\ 0\leq H_{\mathrm{ac},\mathrm{in},t}\leq H_{\mathrm{ac},\mathrm{in},\max} \end{array} \end{array}\right.$$

where *η*_rb,h_ and *η*_ar,c_ are the heat collection efficiency of the waste heat recovery boiler and the cooling efficiency of the adsorption chiller, respectively, *β*_rb,h_ and *β*_ar,c_ are the heating coefficient and the cooling coefficient, respectively, *H*_rh,*t*_ is the heating power on the output side of the waste heat recovery boiler at time *t*, *C*_ac,*t*_ is the cooling power on the output side of the adsorption chiller at time *t*, *H*_rh,in,max_ is the maximum heat collection power allowed on the input side of the waste heat recovery boiler, and *H*_ac,in,max_ is the maximum heat collection power allowed on the input side of the adsorption chiller.

In industrial parks, the thermal power output of HBGT and the corresponding heat transfer balance can be expressed as follows:

(29) $$H_{\mathrm{ht},m,t}=\eta_{\mathrm{re}}P_{\mathrm{ht},m,t},m\in {\hat{L}}_{\mathrm{ht},3}$$

(30) $$\sum_{m\in {\hat{L}}_{\mathrm{ht},3}}H_{\mathrm{ht},m,t}=H_{\mathrm{rh},\mathrm{in},t}+H_{\mathrm{ac},\mathrm{in},t}$$

where *η*_re_ is the HBGT heat conversion efficiency, and $H_{\mathrm{ht},m,t}$ is the heating power of the *m*-th HBGT at time *t*.

(3) Operational Constraints of Electric Boilers and Electric Chillers

Electric boilers and electric chillers use electricity as their input energy source, converting it into heat and cooling, respectively. Their operational constraints ensure that the power consumption does not exceed the allowable maximum values and are defined as follows.

(31) $$\left\{\begin{array}{l} H_{\mathrm{eh},t}=\eta_{\mathrm{eh}}P_{\mathrm{eh},t}\\ C_{\mathrm{ec},t}=\eta_{\mathrm{ec}}P_{\mathrm{ec},t}\\ 0\leq P_{\mathrm{eh},t}\leq P_{\mathrm{eh},\max}\\ 0\leq P_{\mathrm{ec},t}\leq P_{\mathrm{ec},\max} \end{array}\right.$$

where *η*_eh_ is the heating efficiency of the electric boiler, *η*_ec_ is the cooling efficiency of the electric refrigerator, *C*_ec,*t*_ is the cooling power on the output side of the electric refrigerator at time *t*, and *P*_eh,max_ and *P*_ec,max_ are the maximum power consumption allowed by the electric boiler and the electric refrigerator, respectively.

(4) Multi-energy Balance Constraint

The power balance equations for different types of user units are expressed as follows:

(32) $$\begin{array}{l} \sum_{m\in {\hat{L}}_{\mathrm{ht},1}\cup m\in {\hat{L}}_{\mathrm{ht},2}}P_{\mathrm{ht},m,t}+P_{\mathrm{pe},l,t}+P_{v,l,t}^{\mathrm{US}}+\sum_{u\in {\hat{L}}_{\mathrm{es},1}\cup u\in {\hat{L}}_{\mathrm{es},2}}P_{\mathrm{dis},u,t}=P_{\mathrm{load},l,t}^{\mathrm{US}}+\\ \sum_{u\in {\hat{L}}_{\mathrm{es},1}\cup u\in {\hat{L}}_{\mathrm{es},2}}P_{\mathrm{ch},u,t},\;l\in \left\{{\hat{L}}_{\mathrm{us},1},\;{\hat{L}}_{\mathrm{us},2}\right\}\; \end{array}$$

(33) $$\sum_{m\in {\hat{L}}_{\mathrm{ht},3}}P_{\mathrm{ht},m,t}+P_{\mathrm{pe},l,t}+P_{v,l,t}^{\mathrm{US}}+\sum_{u\in {\hat{L}}_{\mathrm{es},3}}P_{\mathrm{dis},u,t}=P_{\mathrm{load},l,t}^{\mathrm{US}}+\sum_{u\in {\hat{L}}_{\mathrm{es},3}}P_{\mathrm{ch},u,t}+P_{\mathrm{eh},t}+P_{\mathrm{ec},t},\;l\in {\hat{L}}_{\mathrm{us},3}$$

(34) $$H_{\mathrm{rh},t}+H_{\mathrm{eh},t}=H_{\mathrm{load},t}$$

(35) $$C_{\mathrm{ec},t}+C_{\mathrm{ac},t}=C_{\mathrm{load},t}$$

where $P_{v,l,t}^{\mathrm{US}}$ is the predicted photovoltaic power of the *l*-th user unit at time *t*, $P_{\mathrm{load},l,t}^{\mathrm{US}}$ is the electrical load of the *l*-th user unit at time *t*, *H*_load,*t*_ and *C*_load,*t*_ are the heat and cold loads of the industrial park at time *t*, respectively.

##### Coupled Operational Constraints

(1) HBGT operational constraints

The operational constraints and boundary conditions of the HBGT are as follows:

(36) $$\lambda_{\mathrm{wh},z,m,t}=\left\{\begin{array}{c} \lambda_{z,m,t},\;0<T_{\mathrm{in},m,t}<T_{\mathrm{com},z}\\ \lambda_{\mathrm{com},z},T_{\mathrm{in},m,t}\geq T_{\mathrm{com},z} \end{array}\right.$$

(37) $$Q_{\mathrm{cab},m,t}=\sum_{z\in {\hat{Z}}_{\mathrm{AG}}}\lambda_{\mathrm{wh},z,m,t}\alpha_{\mathrm{nv},z}N_{\mathrm{in},z,m,t}V_{\mathrm{hg},m,t}$$

(38) $$P_{\mathrm{ht},m,t}=\eta_{c}\sum_{z\in \hat{Z}}\left[\psi_{z}N_{\mathrm{in},z,m,t}^{2}\frac{\rho_{\mathrm{hg},m,t}}{M_{\mathrm{hg},m,t}}\cdot \frac{i_{z}}{2}R\left(T_{\mathrm{com},z}-T_{\mathrm{in},m,t}\right)+\frac{i_{z}}{2}RT_{\mathrm{in},m,t}\lambda_{\mathrm{wh},z,m,t}\psi_{z}N_{\mathrm{in},z,m,t}\right]\cdot V_{\mathrm{hg},m,t}$$

(39) $$\left\{\begin{array}{l} N_{z,i,t}=N_{\mathrm{in},z,m,t}\\ \rho_{i,t}=\rho_{\mathrm{hg},m,t}\\ T_{\mathrm{in},i,t}=T_{\mathrm{in},m,t}\\ \;V_{\mathrm{hg},i,t}=V_{\mathrm{hg},m,t} \end{array}\right.$$

(40) $$0\leq V_{\mathrm{hg},m,t}\leq V_{\mathrm{hg},m,\max}$$

where *m* is the number of HBGTs, *t* is the scheduling time, *λ_z_* is the actual combustion conversion coefficient of gas component *z* before reaching steady-state reaction equilibrium, *λ*_com,*z*_ is the actual combustion conversion coefficient of gas component *z* after reaching steady-state reaction equilibrium, *λ*_wh,*z*_ is the actual combustion conversion coefficient of gas component *z*, *Q*_cab_ is the carbon emission, $\;{\hat{Z}}_{\mathrm{AG}}$ is the set of alkane gas types containing carbon elements in HCNG, *α*_nv,*z*_ is the volume conversion coefficient, *N*_in*,z*_ is the initial molar fraction of gas component *z*, *V*_hg_ is the volume of HCNG consumed by HBGT, *ψ_z_* is the molar fraction conversion coefficient, *η*_c_ is the energy conversion efficiency between mechanical energy and electrical energy, *P*_ht_ is the mixed combustion power generation of HBGT, *ρ*_hg_ is the initial density of HCNG in the combustion chamber, *M*_hg_ is the initial molar mass of HCNG in the combustion chamber, *N_z_*_,*i*,*t*_ is the mole fraction of gas component *z* in gas network node *i* at time *t*, *ρ_i_*_,*t*_ is the HCNG density of gas network node *i* at time *t*, *T*_in,*i*,*t*_ is the temperature of HCNG after pressure regulation in gas network node *i* at time *t*, and *V*_hg,*i*,*t*_ is the volume of HCNG supplied by gas network node *i* at time *t*.

(2) Operational Constraints of Water Electrolysis Units

The hydrogen output flow equation of the water electrolysis unit, its power consumption constraints, and the boundary condition constraints between the water electrolysis unit and the gas network nodes are given as follows:

(41) $$q_{H_{2},n,t}=\frac{\eta_{\mathrm{we}}P_{\mathrm{we},n,t}}{\sigma_{\mathrm{hc}}{\hat{H}}_{\mathrm{gas},z}},\;z\in {\hat{z}}_{H_{2}}$$

(42) $$0\leq P_{\mathrm{we},n,t}\leq P_{\mathrm{we},n,\max}$$

(43) $$q_{H_{2},n,t}=q_{H_{2},i,t}$$

where *η*_we_ is the efficiency of hydrogen production using water electrolysis, and *σ*_hc_ is the correction coefficient of the high calorific value of hydrogen.

(3) Hydrogen storage constraints

The real-time balance relationship of the hydrogen storage tank, the maximum hydrogen storage capacity constraint, and the constraints on the hydrogen inflow and outflow rates to/from the storage unit are expressed as follows:

(44) $$E_{\mathrm{hs},h,t}=E_{\mathrm{hs},h,t-1}+\left(\eta_{\mathrm{hs}}q_{\mathrm{hs},h,t}^{\mathrm{in}}-\frac{q_{\mathrm{hs},h,t}^{\mathrm{out}}}{\eta_{H_{2}}}\right)\Delta t$$

(45) $$0\leq E_{\mathrm{hs},h,t}\leq E_{\mathrm{hs},h,\max}$$

(46) $$\left\{\begin{array}{c} 0\leq q_{\mathrm{hs},h,t}^{\mathrm{in}}\leq U_{\mathrm{hs},h,t}^{\mathrm{in}}q_{\mathrm{hs},h,\max}^{\mathrm{in}}\\ 0\leq q_{\mathrm{hs},h,t}^{\mathrm{out}}\leq U_{\mathrm{hs},h,t}^{\mathrm{out}}q_{\mathrm{hs},h,\max}^{\mathrm{out}}\\ U_{\mathrm{hs},h,t}^{\mathrm{in}}+U_{\mathrm{hs},h,t}^{\mathrm{out}}\leq 1 \end{array}\right.$$

where $\eta_{\mathrm{hs}}$ is the conversion efficiency of the hydrogen storage tank.

### 2.3. Multiple Uncertainty Analysis

#### 2.3.1. Source/Load Side Uncertainty

Considering that there exist varying deviations between the actual and forecasted values of wind power generation and load demand at different time periods, the fluctuation range is described using the ratio between actual and forecasted values. The load demand is mainly divided into urban distribution network load and user-side load, which are formulated as follows:

(47) $$\left\{\begin{array}{c} \Omega_{w,t}=\frac{P_{w,t}}{{\tilde{P}}_{w,t}}\in \left[1-{\hat{\delta }}_{w,t},1+{\hat{\delta }}_{w,t}\right],\;0\leq {\hat{\delta }}_{w,t}\leq 1\\ \Omega_{\mathrm{load},t}^{\mathrm{UD}}=\frac{P_{\mathrm{load},t}^{\mathrm{UD}}}{{\tilde{P}}_{\mathrm{load},t}^{\mathrm{UD}}}\in \left[1-{\hat{\delta }}_{\mathrm{load},t}^{\mathrm{UD}},1+{\hat{\delta }}_{\mathrm{load},t}^{\mathrm{UD}}\right],\;0\leq {\hat{\delta }}_{\mathrm{load},t}^{\mathrm{UD}}\leq 1\\ \Omega_{\mathrm{load},l,t}^{\mathrm{US}}=\frac{P_{\mathrm{load},l,t}^{\mathrm{US}}}{{\tilde{P}}_{\mathrm{load},l,t}^{\mathrm{US}}}\in \left[1-{\hat{\delta }}_{\mathrm{load},l,t}^{\mathrm{US}},1+{\hat{\delta }}_{\mathrm{load},l,t}^{\mathrm{US}}\right],\;0\leq {\hat{\delta }}_{\mathrm{load},l,t}^{\mathrm{US}}\leq 1 \end{array}\right.$$

where ${\hat{\delta }}_{w,t}$ is the power deviation of the *w*-th wind farm at time *t*, ${\hat{\delta }}_{\mathrm{load},t}^{\mathrm{UD}}$ is the load demand deviation of the urban distribution network at time *t*, ${\hat{\delta }}_{\mathrm{load},l,t}^{\mathrm{US}}$ is the load demand deviation of the *l*-th user unit at time *t*, ${\tilde{P}}_{\mathrm{load},t}^{\mathrm{UD}}$ is the predicted value of the urban distribution network load at time *t*, ${\tilde{P}}_{\mathrm{load},l,t}^{\mathrm{US}}$ is the predicted value of the load of the *l*-th user unit at time *t*.

The uncertainties of wind power and load demand exhibit distinct stochastic characteristics, which can be described using either symmetric or asymmetric probability distributions. For example, wind power is directly influenced by wind speed, whose stochastic nature approximately follows a Weibull distribution. In contrast, various types of load demand are typically modeled using a normal distribution [27]. It is worth noting that the parameters involved in the probability density functions can be determined by fitting the historical data of wind speed and load (or load forecast errors).

#### 2.3.2. Combustion Reaction Uncertainty

Considering that the HCNG studied in this paper includes multi-component tracking—mainly methane, ethane, propane, and hydrogen—these components exhibit different combustion characteristics due to variations in heating value and physical properties. However, it is difficult to systematically quantify these combustion characteristics in existing research, which mostly relies on numerical simulations. Moreover, influenced by the internal environmental conditions of the HBGT combustion chamber, the outcome of each combustion reaction within the chamber is subject to variability and randomness [28]. Given the complexity and the number of influencing factors involved in HCNG combustion within HBGT, it is challenging to construct an explicit expression for the combustion reaction.

Therefore, to systematically quantify the uncertainty in combustion reactions, this study applies stochastic optimization theory to approximate the “combustion reaction region” of different gas components in HCNG based on available reference data, thus characterizing the uncertain operational characteristics of HBGT. Assuming that the combustion uncertainty is independent of the HBGT unit index *m*, and considering that the intake temperature of the HBGT determines the combustion process, this section reflects the randomness and fluctuation of the combustion outcome through variations in the actual steady-state combustion conversion coefficient of gas component *z*. Instead of assuming it as a constant, a combustion reaction margin $\vartheta_{\mathrm{sm},z,t}$ is introduced to describe the non-constant steady-state combustion conversion coefficient. Accordingly, the improved calculation formula for the actual combustion conversion coefficient of component *z* is expressed as follows:

(48) $${\hat{\lambda }}_{\mathrm{wh},z,m,t}=\left\{\begin{array}{c} \vartheta_{\mathrm{sm},z,t}\cdot \frac{\lambda_{z,m,t}}{\lambda_{\mathrm{com},z}}\;,\;0<T_{\mathrm{in},m,t}<T_{\mathrm{com},z}\\ \vartheta_{\mathrm{sm},z,t},T_{\mathrm{in},m,t}\geq T_{\mathrm{com},z} \end{array}\right.$$

Additionally, based on existing reference data for combustion reactions, experimental reference values for the steady-state combustion reaction margin of gas component zzz are obtained. However, at different time points, the actual steady-state combustion reaction margin of component *z* may deviate from these experimental reference values. This deviation is described using a fluctuation range, as shown in Equation (49).

(49) $$(1-{\hat{\delta }}_{\mathrm{co},z,t}){\tilde{\vartheta }}_{\mathrm{sm},z,t}\leq \vartheta_{\mathrm{sm},z,t}\leq (1+{\hat{\delta }}_{\mathrm{co},z,t}){\tilde{\vartheta }}_{\mathrm{sm},z,t}$$

where ${\hat{\delta }}_{\mathrm{co},z,t}$ is the deviation of the combustion reaction margin of gas component *z* at time *t*.

**Figure 3:**
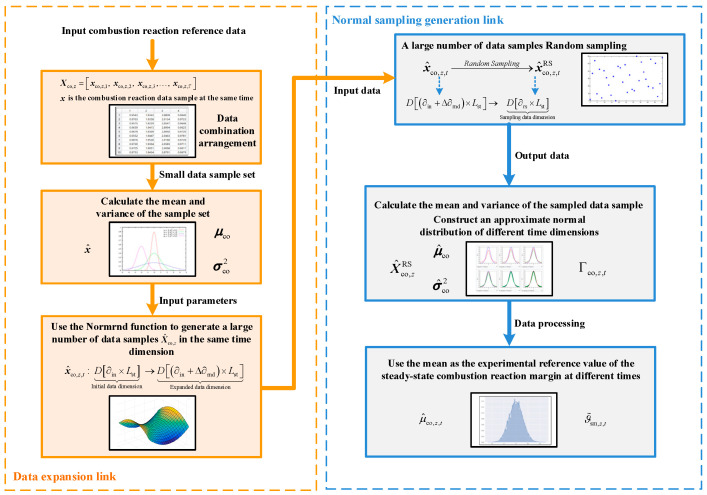
Implementation process of combustion reaction uncertainty description.

The stochastic characteristics of the combustion reaction are assumed to approximately follow a normal distribution. Given the limited number of actual reference data samples within the same time dimension, it is difficult to directly determine the parameters of the probability density function. Therefore, within a reasonable error margin, an approximate normal distribution sampling method based on experimental reference data is employed to expand the dataset. New data sample sets are generated via random sampling, and the mean ${\hat{\mu }}_{\mathrm{co},z,t}$ and variance ${\hat{\sigma }}_{\mathrm{co},z,t}^{2}$ of the corresponding normal distribution $\Gamma_{\mathrm{co},z,t}$ at each time step are determined. The mean ${\hat{\mu }}_{\mathrm{co},z,t}$ is regarded as the experimental reference value for the steady-state combustion reaction margin, the specific implementation process is shown in Figure 3.

#### 2.3.3. Categorical Confidence Interval Description

**Figure 4:**
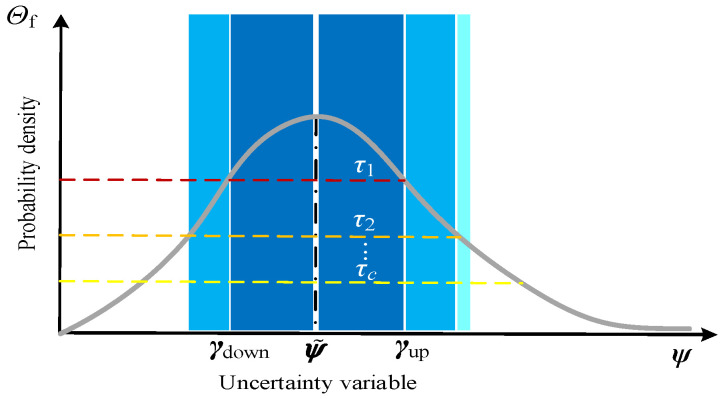
Classification probability confidence intervals for uncertain variables.

Based on the stochastic characteristics of the aforementioned uncertainties, categorical probabilistic confidence intervals can be employed to correct the fluctuation deviations and determine the deviation bounds under different confidence levels. Given a confidence level *τ*, there exist an uncertainty variable matrix ψ and its corresponding predicted (reference) value matrix $\tilde{\psi }$, with the reference value $\tilde{\psi }$ taken as the mean. The probability density function is then used to define the confidence interval boundaries according to different values of *τ_c_*. As illustrated in Figure 4, the categorical confidence interval of the uncertainty variable is bounded by ***γ***_up_ (upper bound) and ***γ***_down_ (lower bound).

As shown in Figure 4, under different confidence levels *τ_c_*, the uncertainty variables may follow symmetric or asymmetric probability distributions due to their distinct stochastic characteristics. Therefore, it is necessary to determine the minimum distance between *γ*_up_, *γ*_down_, and $\tilde{\psi }$ in order to compute the deviation boundaries under multiple uncertainties. The specific calculation formulas are given as follows:

(50) $$\left\{\begin{array}{c} {\hat{\delta }}_{w,t}=\frac{\min\left[{\tilde{P}}_{w,t}-\gamma_{\mathrm{down}}({\tilde{P}}_{w,t},\tau ),\gamma_{\mathrm{up}}({\tilde{P}}_{w,t},\tau )-{\tilde{P}}_{w,t}\right]}{{\tilde{P}}_{w,t}}\\ {\hat{\delta }}_{\mathrm{load},t}^{\mathrm{UD}}=\frac{\min\left[{\tilde{P}}_{\mathrm{load},t}^{\mathrm{UD}}-\gamma_{\mathrm{down}}({\tilde{P}}_{\mathrm{load},t}^{\mathrm{UD}},\tau ),\gamma_{\mathrm{up}}({\tilde{P}}_{\mathrm{load},t}^{\mathrm{UD}},\tau )-{\tilde{P}}_{\mathrm{load},t}^{\mathrm{UD}}\right]}{{\tilde{P}}_{\mathrm{load},t}^{\mathrm{UD}}}\\ {\hat{\delta }}_{\mathrm{load},l,t}^{\mathrm{US}}=\frac{\min\left[{\tilde{P}}_{\mathrm{load},l,t}^{\mathrm{US}}-\gamma_{\mathrm{down}}({\tilde{P}}_{\mathrm{load},l,t}^{\mathrm{US}},\tau ),\gamma_{\mathrm{up}}({\tilde{P}}_{\mathrm{load},l,t}^{\mathrm{US}},\tau )-{\tilde{P}}_{\mathrm{load},l,t}^{\mathrm{US}}\right]}{{\tilde{P}}_{\mathrm{load},l,t}^{\mathrm{US}}}\\ {\hat{\delta }}_{\mathrm{co},z,t}=\frac{\min\left[{\tilde{\vartheta }}_{\mathrm{sm},z,t}-\gamma_{\mathrm{down}}({\tilde{\vartheta }}_{\mathrm{sm},z,t},\tau ),\gamma_{\mathrm{up}}({\tilde{\vartheta }}_{\mathrm{sm},z,t},\tau )-{\tilde{\vartheta }}_{\mathrm{sm},z,t}\right]}{{\tilde{\vartheta }}_{\mathrm{sm},z,t}} \end{array}\right.$$

## 3. SOI-IGDT Considering Exergy Boost

### 3.1. Overview of Exergy Efficiency

Energy is a measure of the state transformation of a system, while exergy, as the usable portion of energy, is a thermodynamic parameter proposed by Rant in 1956. Exergy is defined as the maximum useful work that can be obtained when a system undergoes a reversible thermodynamic process from its initial state to a state in equilibrium with the environment, and it can be regarded as a form of available energy [29]. The stepwise degradation of energy quality in a system can be interpreted as a gradual decline in its ability to perform work.

Since only reversible processes can theoretically achieve complete energy conversion, exergy represents the maximum useful work output or minimum useful work input that can theoretically be achieved. Conversely, the portion of energy that cannot be converted into exergy is referred to as anergy. Any form of energy ${\hat{E}}_{s}$ can be decomposed into exergy ${\hat{E}}_{x}$ and anergy ${\hat{A}}_{n}$, and is mathematically expressed as follows:

(51) $${\hat{E}}_{s}={\hat{E}}_{x}+{\hat{A}}_{n}$$

The exergy of a system can be specifically categorized into input exergy ${\hat{E}}_{x,\mathrm{in}}$ and output exergy ${\hat{E}}_{x,\mathrm{out}}$. The relationship between them can be expressed by the following equation:

(52) $${\hat{E}}_{x,\mathrm{in}}={\hat{E}}_{x,\mathrm{out}}+{\hat{E}}_{x,\mathrm{loss}}$$

where ${\hat{E}}_{x,\mathrm{loss}}$ is the exergy loss in the system.

From the perspective of different energy supply sources, exergy efficiency is adopted as an evaluation metric to quantify the utilization level of useful energy in the overall system or its subsystems. It helps identify weak links in effective energy use and supports the formulation of rational urban integrated energy system planning, operational optimization, and scheduling strategies—ultimately promoting the system’s development toward “high-exergy” performance. The specific expression is defined as follows:

(53) $${\hat{\eta }}_{\mathrm{ex}}=\frac{{\hat{E}}_{x,\mathrm{out}}}{{\hat{E}}_{x,\mathrm{in}}}$$

For the optimization scheduling of EHUIES considering multiple uncertainties, the system output exergy reflects the energy utilization on the user side, while the system input exergy represents the energy supply on the supply side. A higher exergy efficiency indicates a lower proportion of exergy loss relative to the input exergy during the supply process, suggesting that the system is closer to achieving high-exergy performance.

### 3.2. Overview of SOI-IGDT

The traditional IGDT does not account for the probability distributions of uncertain variables, and instead only considers the maximum fluctuation range within the [0, 1] interval. This leads to a coarse and oversimplified characterization of uncertainty. Moreover, it applies the same robustness coefficient *ζ* across all time periods as the boundary condition for optimization, which results in a “temporal symmetry of deviation.” This assumption is rather idealized and may deviate significantly from the actual fluctuation intervals. As demonstrated in Section 2.3.3 on the classification of confidence intervals for multiple uncertainties, the probabilistic distribution characteristics of uncertainties at different time periods can constrain the effective fluctuation range under traditional IGDT. Based on this, the conventional IGDT exhibits the following limitations:

(1) The deviation factor in traditional IGDT requires setting based on the decision-maker’s experience, which is highly subjective and lacks risk assessment of the model optimization results.
(2) When considering the stochastic characteristics of multiple uncertainties, the maximum fluctuation range obtained using traditional IGDT optimization tends to be overly conservative and may not accurately reflect the actual fluctuation range.
(3) The traditional IGDT’s maximum boundary condition is linearly consistent (symmetric) across time, using a global deviation parameter ζ as the optimization target. This leads to an overly conservative robust solution that lacks effective integration with actual uncertainty fluctuations. Moreover, the impact of maximum deviations on total system cost varies over time, causing inconsistency in the objective function’s direction. When actual uncertainty bounds at different times are known, using the original ζ as the optimization target may result in infeasibility, increased nonlinearity, and reduced solution reliability and efficiency.

Therefore, to address the above shortcomings when considering the stochastic characteristics of multiple uncertainties, this section proposes a SOI-IGDT by integrating IGDT with stochastic optimization theory. The specific improvements are as follows:

(1) First, by applying chance constraints from stochastic optimization theory, the traditional IGDT deviation factor is removed. The constraint satisfaction probability *Pr* is guaranteed to be no less than the given confidence level *τ*. Thus, the original IGDT constraint on the optimization objective can be reformulated as follows:

(54) $$\max F(\hat{\kappa },\psi )\leq {\hat{F}}_{\mathrm{et}}=(1+\phi_{d})F_{\mathrm{ac}}\to \mathrm{Pr}\left\{\max F(\hat{\kappa },\psi )\leq F_{\mathrm{ac}}\right\}\geq \tau$$

Accordingly, considering the stochastic characteristics of multiple uncertainties, the actual boundary conditions of the uncertainty variables ψ need to be classified and adjusted based on the confidence level *τ*. Combining the content from Section 2.3.3 and using the boundary-solving method in Equation (50), the actual boundary conditions of multiple uncertainties are corrected as follows:

(55) $$\psi \in \hat{\Phi }\left(\tilde{\psi },\tau \right)=\left\{\left.\psi \right|\psi^{-}\left(\tilde{\psi },\tau \right)\leq \psi \leq \psi^{+}\left(\tilde{\psi },\tau \right)\right\}$$

where $\hat{\Phi }$ is the corrected confidence interval of the uncertainty variable, $\psi^{-}$ is the lower bound of the corrected confidence interval, and $\psi^{+}$ is the upper bound of the corrected confidence interval.

(2) Secondly, the original deviation ζ is a global optimization variable with consistent boundary conditions across all time periods, only satisfying a unified maximum deviation. This approach is unsuitable for scenarios where uncertainty varies over time. Since deviations at different times impact the system differently, the optimization must consider the time-varying effects of deviations on total system cost. Therefore, the model accounts for the impact of actual deviations at each time step during optimization. Furthermore, under multiple uncertainties, it is necessary to decompose multiple objective deviations by time periods and establish an uncertainty deviation matrix ${\hat{\zeta }}_{r}$ with time scale *t*. Using the time-varying deviation $\zeta_{r,t}$ allows better tracking of deviation boundary changes considering the uncertainty probability distributions at different times. The improved uncertainty variable fluctuation range is expressed as follows:

(56) $${\hat{\zeta }}_{r}=\left[\zeta_{r,1},\zeta_{r,2},\ldots ,\zeta_{r,t}\right],\;\forall r\in \left[1,R_{\mathrm{uv}}\right],\;t\in \left[1,T_{\mathrm{vd},r}\right]$$

(57) $$\psi \in \hat{\Phi }\left(\tilde{\psi },\tau \right)=\left\{\left.\psi \right|\psi^{-}\left(\tilde{\psi },\tau \right)\leq \psi \leq \psi^{+}\left(\tilde{\psi },\tau \right)\right\}$$

where $\hat{\zeta }$ is the time-varying deviation matrix for multiple uncertainties, *R_uv_* is the number of uncertainty variables, *T*_vd,*r*_ is the effective time period of the forecast (reference) value for the *r*-th uncertainty variable, determined by whether its value is zero at time *t*.

Uncertainty probability distributions affect the variation of deviation boundaries at different time points, making it difficult to accurately determine both boundaries of the fluctuation interval. Therefore, a time-varying deviation direction indicator is introduced into the constraints to reflect the actual fluctuation direction of BBB around AAA at each time, eliminating the subjective assumption of a single deviation direction in traditional IGDT. In practice, while managing uncertainty risks and satisfying Equation (54), the maximum fluctuation intervals obtained at different times exhibit mutual coordination, avoiding the one-sided degradation observed in conventional IGDT. To capture the chance-constrained risk-averse feature, based on the expected cost under chance constraints, a relaxed directional condition for uncertainty fluctuation intervals at each time is established. This formulates a deviation maximization problem that ensures the expected cost does not exceed a given threshold at a specified confidence level, thereby removing the max operator in Equation (54) and expanding the solution space of the model. The formulation is expressed as follows:

(58) $$\left\{\begin{array}{l} \left({\hat{u}}_{+}-{\hat{u}}_{-}\right)\psi +\left({\hat{u}}_{+}-{\hat{u}}_{-}\right)\tilde{\psi }=\hat{\zeta }⊙\tilde{\psi }\\ \hat{\chi }=\mathrm{sign}\left(\psi -\tilde{\psi }\right),\;{\hat{u}}_{+}=\frac{\left|\hat{\chi }\right|+\hat{\chi }}{2},\;{\hat{u}}_{-}=\frac{\left|\hat{\chi }\right|-\hat{\chi }}{2} \end{array}\right.$$

where $\hat{\chi }$, ${\hat{u}}_{+}$, and ${\hat{u}}_{-}$ denote the directional indicator matrix of uncertainty fluctuations, the positive direction indicator, and the negative direction indicator, respectively. ⊙ represents the Hadamard product operator (element-wise matrix multiplication).

(3) In traditional IGDT, the optimization of multiple uncertainties relies on subjectively assigned priority levels, aiming to maximize deviation under idealized conditions without considering the actual probability distributions. However, in practical system optimization, multiple uncertainties are often correlated and constrained by system balance and equipment coupling. When the true probabilistic characteristics are considered, the feasible region is further restricted by the adjusted confidence intervals. As these intervals vary significantly over time, the predefined global priority scheme may hinder the optimization process. Therefore, without incorporating the actual distributions of uncertainties, the traditional IGDT approach tends to be overly conservative. Therefore, based on the specific improvements introduced in Step (2), $\forall r\in \left[1,R_{\mathrm{uv}}\right]$ , vor the time-varying deviation matrix ${\hat{\zeta }}_{r}$ of the *r*-th uncertainty variable, the time-varying deviations $\zeta_{r,t}$ at different time steps are simultaneously driven toward maximization. Meanwhile, the priority order of ${\hat{\zeta }}_{r}$ will be determined within the optimization objective to better reflect real-world conditions. Therefore, in this section, the ∞-norm of the matrix is employed in the optimization objective to achieve maximization. By approximating the mean of time-varying deviations over their effective time periods, this approach ensures consistency with the form of the original optimization objective. In addition, a reciprocal matrix ${\tilde{T}}_{\mathrm{vd}}={\left[1/T_{\mathrm{vd},1},1/T_{\mathrm{vd},2},\ldots ,1/T_{\mathrm{vd},r}\right]}^{Τ}$ of the effective time periods for multiple uncertainties is constructed, and the improved optimization objective is then formulated as follows:

(59) $$\underset{\hat{\kappa }}{\max}\;\zeta_{\mathrm{CM}}={\left\|\hat{H}\right\|}_{\infty },\hat{H}=\hat{\zeta }⊙{\tilde{T}}_{\mathrm{vd}}$$

where *ζ_CM_* is the actual classified mean deviation of the uncertainty variables, and $\hat{H}$ is the time-varying mean judgment matrix of multiple uncertainties.

Therefore, the system optimization model based on SOI-IGDT is specifically formulated as follows:

(60) $$\left\{\begin{array}{c} \underset{\hat{\kappa }}{\max}\;\zeta_{\mathrm{CM}}={\left\|\hat{H}\right\|}_{\infty },\hat{H}=\hat{\zeta }⊙{\tilde{T}}_{\mathrm{vd}}\\ \;s.t.\;\mathrm{Pr}\left\{F(\hat{\kappa },\psi )\leq F_{\mathrm{ac}}\right\}\geq \tau \\ \hat{h}(\hat{\kappa },\psi )=0,\;\hat{g}(\hat{\kappa },\psi )\leq 0\\ \psi \in \hat{\Phi }\left(\tilde{\psi },\tau \right)=\left\{\left.\psi \right|\psi^{-}\left(\tilde{\psi },\tau \right)\leq \psi \leq \psi^{+}\left(\tilde{\psi },\tau \right)\right\}\\ \;\left({\hat{u}}_{+}-{\hat{u}}_{-}\right)\psi +\left({\hat{u}}_{+}-{\hat{u}}_{-}\right)\tilde{\psi }=\hat{\zeta }⊙\tilde{\psi }\\ \hat{\chi }=\mathrm{sign}\left(\psi -\tilde{\psi }\right),\;{\hat{u}}_{+}=\frac{\left|\hat{\chi }\right|+\hat{\chi }}{2},\;{\hat{u}}_{-}=\frac{\left|\hat{\chi }\right|-\hat{\chi }}{2} \end{array}\right.$$

### 3.3. System Optimization Scheduling Model Based on SOI-IGDT

Based on the aforementioned basic structure and optimization model of EHUIES, this section establishes an EHUIES optimization scheduling model considering multiple uncertainties, based on SOI-IGDT. The model primarily includes the optimization objective and the corresponding constraints.

(1) Optimization Objective

Combining the multi-uncertainty analysis in Section 2.3, ψ considers the uncertainties and corresponding stochastic characteristics of wind power, load, and combustion reactions. Additionally, the effective time period for wind power, load, and combustion reactions is set as *T*, i.e., $T_{\mathrm{vd},r}=T$. Therefore, the system model’s optimization objective can be expressed as follows:

(61) $$\underset{\hat{\kappa }}{\max}\;\zeta_{\mathrm{CM}}={\left\|\hat{H}\right\|}_{\infty },\;\hat{H}=\hat{\zeta }⊙{\tilde{T}}_{\mathrm{vd}},\;\hat{\zeta }=\left[\begin{array}{c} {\hat{\zeta }}_{w}\\ {\hat{\zeta }}_{\mathrm{load}}^{\mathrm{UD}}\\ {\hat{\zeta }}_{\mathrm{load},l}^{\mathrm{US}}\\ {\hat{\zeta }}_{\mathrm{co},z} \end{array}\right]$$

where ${\hat{\zeta }}_{w}$, ${\hat{\zeta }}_{\mathrm{load}}^{\mathrm{UD}}$, ${\hat{\zeta }}_{\mathrm{load},l}^{\mathrm{US}}$, and ${\hat{\zeta }}_{\mathrm{co},z}$ represent the time-varying deviation matrices of the actual power output of the *w*-th wind farm, the urban distribution network load, the load of the *l*-th user unit, and the time-varying deviation matrix of the actual steady-state combustion reaction margin of gas component *z*, respectively.

(2) Constraints

By setting the confidence level *τ*, more accurate and reasonable scheduling strategies can be formulated for the EHUIES optimal operation. Here, $F_{\mathrm{ac}}$ represents the deterministic model solution results, and $\psi ={\left[P_{w},P_{\mathrm{load}}^{\mathrm{UD}},P_{\mathrm{load},l}^{\mathrm{US}},\vartheta_{\mathrm{sm},z}\right]}^{Τ}$ is established as time-dependent with respect to the scheduling horizon. Accordingly, the system’s total operation cost risk assessment constraint based on classified confidence can be expressed as follows.

(62) $$\mathrm{Pr}\left\{F(\hat{\kappa },P_{w},P_{\mathrm{load}}^{\mathrm{UD}},P_{\mathrm{load},l}^{\mathrm{US}},\vartheta_{\mathrm{sm},z})\leq F_{\mathrm{ac}}\right\}\geq \tau$$

The boundary conditions for multiple uncertain variables can be specifically expressed by the following formula:

(63) $$\left\{\begin{array}{l} \left({\hat{u}}_{+}-{\hat{u}}_{-}\right)\psi +\left({\hat{u}}_{+}-{\hat{u}}_{-}\right)\tilde{\psi }=\hat{\zeta }⊙\tilde{\psi },\tilde{\psi }={\left[{\tilde{P}}_{w},{\tilde{P}}_{\mathrm{load}}^{\mathrm{UD}},{\tilde{P}}_{\mathrm{load},l}^{\mathrm{US}},{\tilde{\vartheta }}_{\mathrm{sm},z}\right]}^{Τ}\\ \hat{\chi }=\mathrm{sign}\left(\psi -\tilde{\psi }\right),\;{\hat{u}}_{+}=\frac{\left|\hat{\chi }\right|+\hat{\chi }}{2},\;{\hat{u}}_{-}=\frac{\left|\hat{\chi }\right|-\hat{\chi }}{2} \end{array}\right.$$

**Figure 5:**
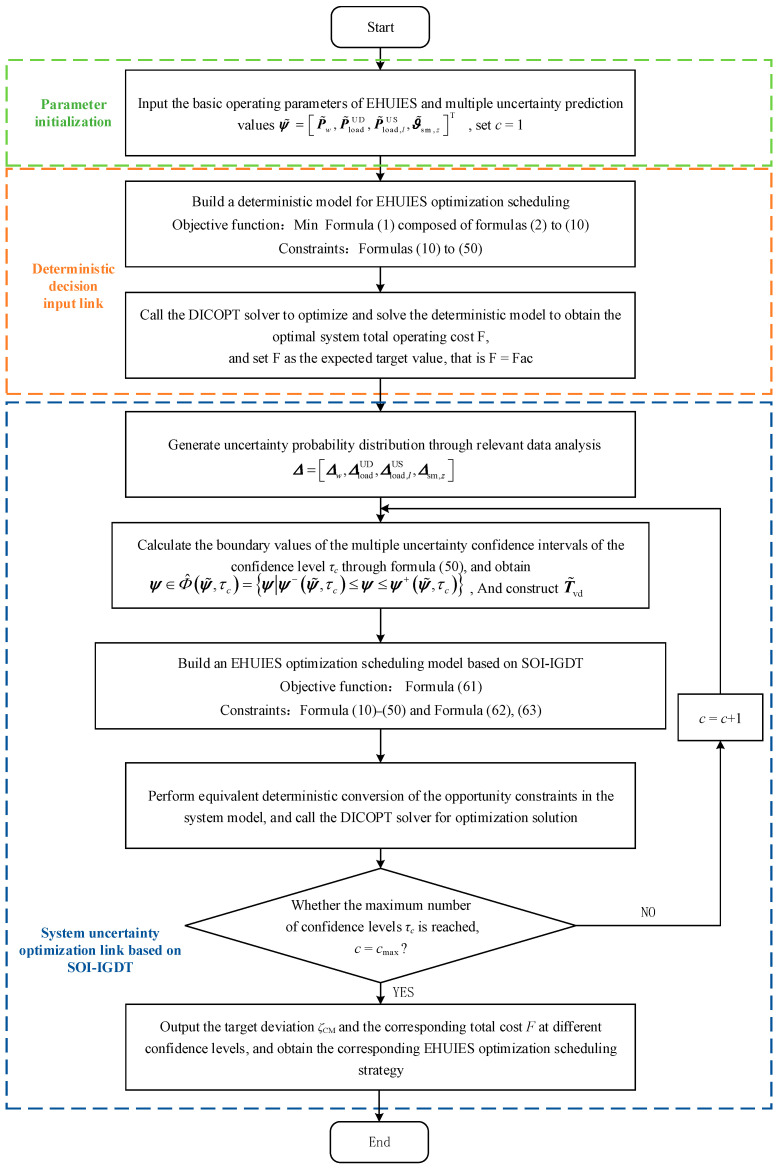
Flowchart of optimal scheduling of EHUIES based on SOI-IGDT.

In addition, other related EHUIES constraints also include those from Equations (11) to (50). In summary, $\forall \tau_{c}\in \left[{\hat{\tau }}_{\min},{\hat{\tau }}_{\max}\right]$, $c\in {\mathbb{Z}}_{+}$, the EHUIES optimization scheduling process based on SOI-IGDT is shown in Figure 5.

### 3.4. Model Solution of SOI-IGDT Considering Exergy Boost

The previous sections have introduced the definition of exergy efficiency, the formulation of the SOI-IGDT framework, and its application to the scheduling optimization model of EHUIES under uncertainty. Building upon this foundation, this section aims to incorporate an exergy enhancement mechanism to further improve the system’s thermodynamic performance. Specifically, a penalty-based approach is designed to embed exergy efficiency improvement into the original model. The motivation for introducing exergy-based optimization lies in the need to improve the quality of energy utilization in integrated energy systems. While traditional scheduling approaches often focus on energy quantity and cost minimization, they tend to overlook energy grade differences among electricity, heat, cold, and gas. Enhancing exergy efficiency allows for a more refined and effective use of energy resources, which is essential for achieving high-performance, low-carbon multi-energy system operation.

To achieve efficient utilization of electricity, cooling, heating, HCNG, and other energy forms in EHUIES, this section introduces an exergy efficiency evaluation indicator into the aforementioned model to further enhance the system’s exergy. However, since the existing SOI-IGDT does not effectively facilitate this process, improvements to the original SOI-IGDT are necessary. The existing approach for handling the exergy efficiency optimization objective under multi-objective optimization frameworks based on IGDT and stochastic optimization incorporates the maximization of exergy efficiency from the deterministic model into the constraints. Specifically, it ensures that, under different confidence levels, the exergy efficiency ${\hat{\eta }}_{x}$ is not lower than the deterministic model’s exergy efficiency ${\hat{\eta }}_{\mathrm{ex},\mathrm{ac}}$. Specifically, when $\forall \tau_{c}\in \left[{\hat{\tau }}_{\min},{\hat{\tau }}_{\max}\right]$, it follows that ${\hat{\eta }}_{\mathrm{ex}}\geq {\hat{\eta }}_{\mathrm{ex},\mathrm{ac}}$ holds, thereby ensuring that the system exergy efficiency does not fall below the expected target value. It is evident that the feasible solution space for exergy efficiency optimization depends on both ${\hat{\eta }}_{\mathrm{ex},\mathrm{ac}}$ and $F(\hat{\kappa },\psi )$. However, since the primary optimization objective is to maximize the deviation margin, this does not necessarily guarantee condition ${\hat{\eta }}_{\mathrm{ex}}\gg {\hat{\eta }}_{\mathrm{ex},\mathrm{ac}}$, and the resulting ${\hat{\eta }}_{\mathrm{ex}}$ will represent a compromised solution after system optimization. Consequently, in the process of pursuing maximum exergy enhancement, the aforementioned method may exhibit randomness and limitations. Based on the aforementioned limitations, the following improvement strategies are proposed:

(1) The exergy efficiency is incorporated as an evaluation metric within the constraint framework, replacing the expected target value ${\hat{\eta }}_{\mathrm{ex},\mathrm{ac}}$ derived from the deterministic model with a new assessment target ${\hat{\eta }}_{\mathrm{ex},\mathrm{st}}$. During the optimization process, the discrepancy between the actual exergy performance ${\hat{\eta }}_{\mathrm{ex}}$ and the target value ${\hat{\eta }}_{\mathrm{ex},\mathrm{ac}}$ is analyzed. A penalty function is constructed in the form of a first-order term using the method of Lagrange multipliers, while the second-order term is neglected to reduce computational complexity. Through iterative updates, this penalty mechanism gradually minimizes the deviation between ${\hat{\eta }}_{\mathrm{ex}}$ and ${\hat{\eta }}_{\mathrm{ex},\mathrm{ac}}$, thereby enhancing the overall system exergy efficiency ${\hat{\eta }}_{x}$.
(2) Due to the interdependent relationship between ${\hat{\eta }}_{\mathrm{ex}}$ and $F(\hat{\kappa },\psi )$ within the system, enhancing ${\hat{\eta }}_{x}$ induces corresponding adjustments in $F(\hat{\kappa },\psi )$, depending on the variation direction of ${\hat{\eta }}_{x}$ during the iterative process. Therefore, in the constraint formulation of $F(\hat{\kappa },\psi )$ after equivalent deterministic transformation, a variation multiplier is introduced to dynamically track the iterative evolution of ${\hat{\eta }}_{\mathrm{ex}}$.

Firstly, the energy utilization within all regions of the EHUIES is analyzed, which primarily consists of the urban distribution network, the HCNG network, and urban terminal user units. To ensure consistency in the representation of variables, all energy forms are expressed in power units. A detailed examination of the input–output energy forms across different EHUIES regions reveals that the input side mainly includes natural gas sources, wind farms, photovoltaic power stations, user-side PV generation, electricity purchased by users, and discharging from electrical energy storage systems. The output side primarily comprises urban grid electrical loads, user-side electrical loads, energy storage charging, industrial park cooling loads, industrial park heating loads, and HCNG network gas loads. Accordingly, the exergy efficiency of the system can be expressed as follows:

(64) $${\hat{\eta }}_{\mathrm{ex}}=\frac{\sum_{t=1}^{T}\left[\varpi_{e}P_{\mathrm{load},t}^{\mathrm{UD}}+\sum_{l=1}^{L}\varpi_{e}P_{\mathrm{load},l,t}^{\mathrm{US}}+\sum_{u=1}^{U}\varpi_{e}P_{\mathrm{ch},u,t}+\varpi_{c}C_{\mathrm{load},t}+\varpi_{h}H_{\mathrm{load},t}+\sum_{i=1}^{I}\varpi_{g}E_{d,i,t}\right]}{\sum_{t=1}^{T}\left[\sum_{s=1}^{S}\varpi_{g}q_{s,t}{\hat{H}}_{\mathrm{ng},s}+\sum_{w=1}^{W}\varpi_{e}P_{w,t}+\varpi_{e}P_{\mathrm{vps},t}^{\mathrm{UD}}+\sum_{l=1}^{L}\varpi_{e}\left(P_{v,l,t}^{\mathrm{US}}+P_{\mathrm{ep},l,t}\right)+\sum_{u=1}^{U}\varpi_{e}P_{\mathrm{dis},u,t}\right]}$$

where $\varpi_{e}$, $\varpi_{c}$, $\varpi_{h}$, and $\varpi_{g}$ represent the energy quality coefficients of electricity, cooling, heating, and gas, respectively.

Secondly, the exergy efficiency objective based on the multiplier method is incorporated into the constraint set of the EHUIES optimal scheduling model. Adjustment multipliers, namely the total cost adjustment multiplier ${\hat{\gamma }}_{\mathrm{tc}}$ and the exergy efficiency deviation multiplier ${\hat{\beta }}_{\mathrm{ex}}$, are introduced on the right-hand side of the inequality constraints for the system total operating cost and exergy efficiency, respectively. The specific formulation is expressed as follows:

(65) $$\left\{\begin{array}{c} F\left[\hat{\kappa },{\tilde{\Delta }}_{w}(\tau ),{\tilde{\Delta }}_{\mathrm{load}}^{\mathrm{UD}}(\tau ),{\tilde{\Delta }}_{\mathrm{load},l}^{\mathrm{US}}(\tau ),{\tilde{\Delta }}_{\mathrm{sm},z}(\tau )\right]\leq {\hat{\gamma }}_{\mathrm{tc}}F_{\mathrm{ac}}\\ {\hat{\beta }}_{\mathrm{ex}}\left({\hat{\eta }}_{\mathrm{ex},\mathrm{st}}-{\hat{\eta }}_{\mathrm{ex}}\right)\leq \varepsilon_{\mathrm{ex}} \end{array}\right.$$

where $\varepsilon_{\mathrm{ex}}$ is the deviation accuracy of the exergy efficiency.

Finally, the convergence criteria and the corresponding multiplier update rules need to be determined. The iterative residuals of the system total operation cost and exergy efficiency are used as the convergence conditions, which can be expressed as follows:

(66) $$\left|{\hat{\eta }}_{\mathrm{ex}}^{\left(k\right)}-{\hat{\eta }}_{\mathrm{ex}}^{\left(k-1\right)}\right|\leq \varepsilon_{r1}$$

(67) $$\left|\frac{F^{\left(k\right)}\left[\hat{\kappa },{\tilde{\Delta }}_{w}(\tau ),{\tilde{\Delta }}_{\mathrm{load}}^{\mathrm{UD}}(\tau ),{\tilde{\Delta }}_{\mathrm{load},l}^{\mathrm{US}}(\tau ),{\tilde{\Delta }}_{\mathrm{sm},z}(\tau )\right]-F^{\left(k-1\right)}\left[\hat{\kappa },{\tilde{\Delta }}_{w}(\tau ),{\tilde{\Delta }}_{\mathrm{load}}^{\mathrm{UD}}(\tau ),{\tilde{\Delta }}_{\mathrm{load},l}^{\mathrm{US}}(\tau ),{\tilde{\Delta }}_{\mathrm{sm},z}(\tau )\right]}{F^{\left(k\right)}\left[\hat{\kappa },{\tilde{\Delta }}_{w}(\tau ),{\tilde{\Delta }}_{\mathrm{load}}^{\mathrm{UD}}(\tau ),{\tilde{\Delta }}_{\mathrm{load},l}^{\mathrm{US}}(\tau ),{\tilde{\Delta }}_{\mathrm{sm},z}(\tau )\right]}\right|\leq \varepsilon_{r2}$$

where *ε*_r1_ and *ε*_r2_ denote the iterative convergence tolerances for exergy efficiency and system total operation cost, respectively; *k* is the iteration count.

If the convergence conditions in Equations (66) and (67) are not simultaneously satisfied, the multiplier update rules for ${\hat{\gamma }}_{\mathrm{tc}}$ and ${\hat{\beta }}_{\mathrm{ex}}$ need to be applied, as specified in Equation (68). Since the iterative residuals are used as the basis for multiplier updates, initial conditions prior to iteration (i.e., at iteration 0) must be set. The exergy efficiency and system total operation cost ${\hat{\eta }}_{\mathrm{ex},\mathrm{cd}}$ and $F_{\mathrm{cd}}$ obtained from the optimization in Section 3.3 are used as references to establish ${\hat{\eta }}_{\mathrm{ex}}^{\left(0\right)}={\hat{\eta }}_{\mathrm{ex},\mathrm{cd}}$ and $F^{\left(0\right)}=F_{\mathrm{cd}}$ under different confidence levels.

(68) $$\left\{\begin{array}{l} {\hat{\gamma }}_{\mathrm{tc}}^{\left(k\right)}={\hat{\gamma }}_{\mathrm{tc}}^{\left(k-1\right)}+\varphi_{d}\left({\hat{\eta }}_{\mathrm{ex}}^{\left(k-1\right)}-{\hat{\eta }}_{\mathrm{ex}}^{\left(k-2\right)}\right)\\ {\hat{\beta }}_{\mathrm{ex}}^{\left(k\right)}={\hat{\beta }}_{\mathrm{ex}}^{\left(k-1\right)}+\omega_{d}^{2}\left({\hat{\eta }}_{\mathrm{ex},\mathrm{st}}-{\hat{\eta }}_{\mathrm{ex}}^{\left(k-1\right)}\right) \end{array}\right.,\;k\geq 2$$

where both $\varphi_{d}$ and $\omega_{d}$ are positive constants introduced to accelerate the convergence of the proposed method, where $3<\omega_{d}<6$ and $0<\varphi_{d}<2$.

**Figure 6:**
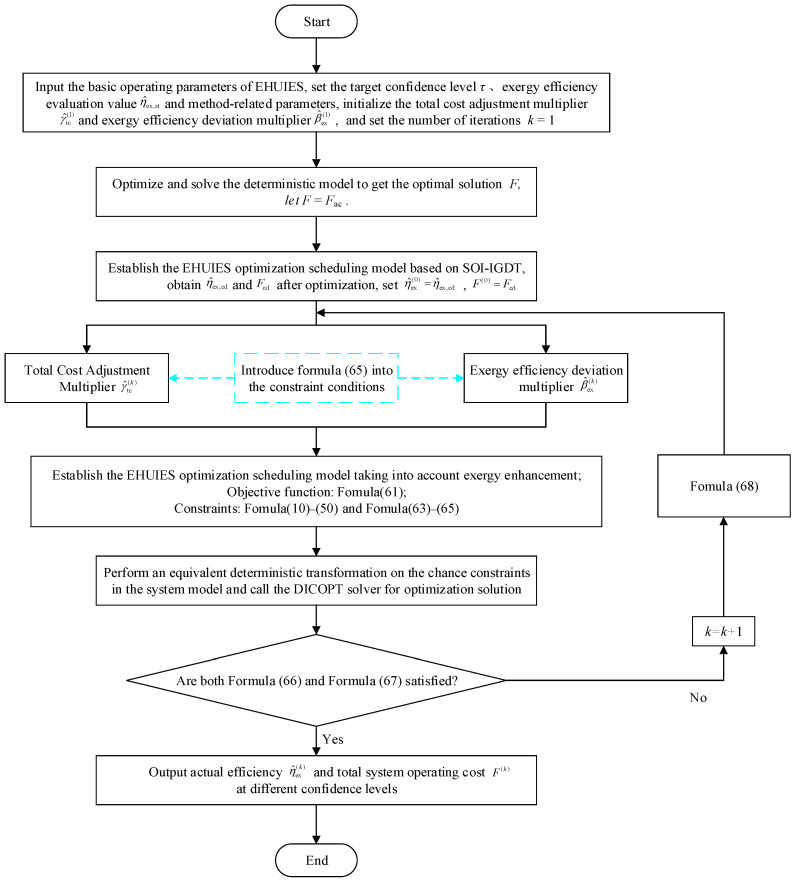
Solution process of SOI-IGDT considering exergy improvement.

Based on Figure 5, Figure 6 presents the solution process of the SOI-IGDT considering exergy enhancement. The deterministic decision input step remains unchanged, as it is still necessary to determine the expected target value *F*_ac_ through a deterministic model to serve as an input condition for system optimization scheduling.

## 4. Results and Discussion

**Figure 7:**
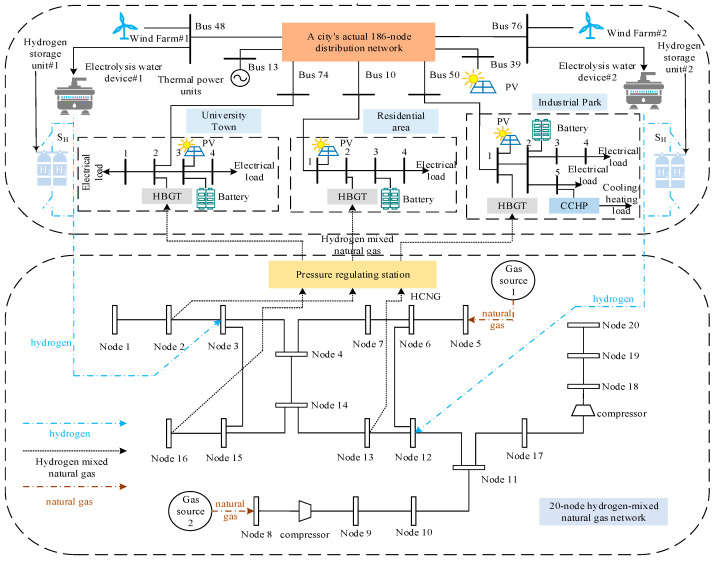
Structure diagram of a 186-bus actual urban distribution network coupled with a 20-node HCNGN.

In this section, a case study that was conducted using a practical 186-bus urban distribution network system coupled with a 20-node HCNGN to verify the effectiveness of the proposed method and model is discussed. The structure of the system is illustrated in Figure 7. The system’s dynamic economic dispatch period is set to 24 h, and $\forall \tau \in \left[0.75,\;0.95\right]$. The initial values of the total cost adjustment multiplier ${\hat{\gamma }}_{\mathrm{tc}}^{\left(0\right)}$ and the exergy efficiency deviation multiplier ${\hat{\beta }}_{\mathrm{ex}}^{\left(0\right)}$ are set to 1 and 2, respectively. The convergence tolerances *ε*_r1_ and *ε*_r2_ are both set to 10^−3^. The stochastic characteristics of combustion reaction uncertainty are described using extended data dimensions $D\left[\left(\partial_{\mathrm{in}}+\Delta \partial_{\mathrm{rnd}}\right)\times L_{\mathrm{st}}\right]=100$ and sampled data dimensions $D\left[\partial_{\mathrm{rs}}\times L_{\mathrm{st}}\right]=50$, and the parameters of the probability distribution are obtained using the method described in Section 2.3.2. In addition, the uncertainties of cooling, heating, and gas loads are temporarily neglected. The wind curtailment cost is not considered in the deterministic optimization model. The corresponding load values are redefined, and a deterministic optimization is carried out to obtain a total system cost of $F_{\mathrm{ac}}=1442.32$ (ten thousand yuan), with $\mu_{\mathrm{hs}}=0$. The exergy efficiency evaluation value is then given as ${\hat{\eta }}_{\mathrm{ex},\mathrm{st}}=96.5\%$.

**Figure 8:**
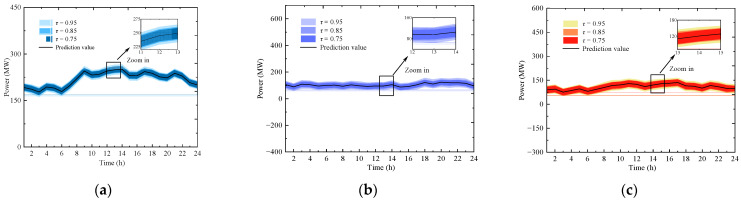
(**a**) Curve band in electric load of urban distribution network under different confidence levels; (**b**) curve band in predicted power of wind farm #1 under different confidence levels; (**c**) curve band in predicted power of wind farm #2 under different confidence levels.

This section adopts confidence levels $\tau \in \left\{0.75,\;0.85,\;0.95\right\}$ as scenario classifications, and derives the corresponding uncertainty probability distributions based on relevant real data statistics. Specifically, Figure 8 illustrates the forecast intervals for urban distribution network electrical load and wind farms under different confidence levels. The description methods for other uncertainties are similar to those in Figure 8 and are therefore omitted here. Based on these varying forecast intervals, the classification confidence descriptions for multiple uncertainties such as wind power, load, and combustion reactions are obtained using the method from Section 2.3.3. This yields the corrected confidence intervals for the urban distribution network, residential communities, university campuses, industrial parks’ electrical loads, wind farms, and the combustion reactions of methane, ethane, propane, and hydrogen.

### 4.1. Uncertainty Optimal Scheduling Strategy for Electric–Hydrogen–Gas Hybrid Urban Integrated Energy System

Based on the previously described structure of the EHUIES, this section sets up the following four cases to analyze the impact of the stochastic characteristics of multiple uncertainties on the optimal scheduling of EHUIES:

- Case 1: A modified confidence interval representing multiple uncertainties with a confidence level of *τ* = 0.75.
- Case 2: A modified confidence interval representing multiple uncertainties with a confidence level of *τ* = 0.85.
- Case 3: A modified confidence interval representing multiple uncertainties with a confidence level of *τ* = 0.95.
- Case 4: A deterministic optimization model without considering changes in the confidence level *τ*.

It should be noted that the effectiveness of the proposed optimal scheduling strategy will be evaluated from the following aspects: the actual fluctuation of the wind farm, electricity, and HCNG purchases by user units, operation and physical characteristics of the HCNGN, steady-state combustion margin of HCNG, total system operating cost, and the actual category-wise normalized deviation.

(1) Actual Fluctuation Characteristics of the Wind Farm

**Figure 9:**
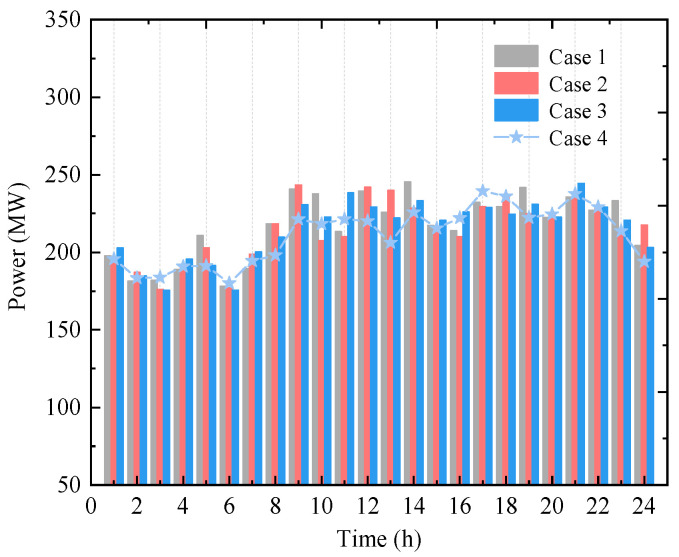
Change of wind power consumption in Cases 1–4.

All wind farm forecast power deviations exhibit a bidirectional characteristic, fluctuating both above and below the forecasted values. In this section, the analysis focuses on the scenario where hydrogen blending via P2G is considered and the SH unit is disconnected. Figure 9 illustrates the temporal variation of the actual total wind power output under different scenarios.

As shown in Figure 9, when wind power uncertainty information is incorporated, variations in the confidence level τ lead to differences in the actual total wind power output across Cases 1–3 compared to Case 4 at various time points. Overall, Case 1 shows actual wind power outputs that are closer to the forecasted values compared to Cases 2–4.

(2) Electricity and HCNG Purchase Behavior of User Units

**Figure 10:**
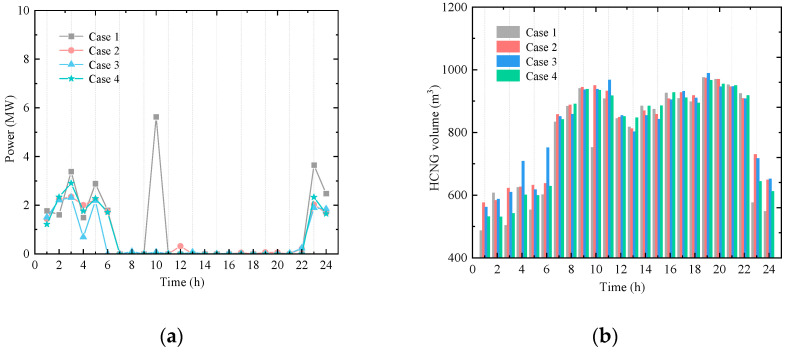
(**a**) Electricity purchase of user units in Cases 1–4; (**b**) HCNG purchase of user units in Cases 1–4.

To analyze the impact of multiple uncertainties on the economic performance of the user-side energy input, Figure 10 presents the total electricity and HCNG purchases by all user units.

To address the impact of multiple uncertainties on the user-side energy procurement strategy, Figure 10 illustrates the total electricity and HCNG purchases by all user units. It can be observed that, in response to load uncertainty at different time points, user units tend to prioritize HCNG purchases to support HBGT power generation, with electricity purchases serving as a supplementary measure. As the confidence level τ increases, the fluctuation range of uncertainties expands. The proposed model yields energy procurement strategies (Cases 1–3) that meet user demands under varying scenarios, showing notable differences from the deterministic Case 4. In Figure 10a, under *τ* = 0.75, the narrower uncertainty range leads to more electricity purchases at certain times to mitigate wind and load uncertainty impacts. Correspondingly, as shown in Figure 10b, HCNG purchases are equal to or slightly lower than those in Case 4. When *τ* reaches 0.95, the deviation in electricity purchases from Cases 1 and 2 becomes significant, prompting a substantial increase in HCNG procurement at certain time intervals.

Influenced by time-of-use electricity pricing and HCNG price incentives, the electricity and HCNG procurement costs for user units under Cases 1–4 are summarized in Table 1.

As shown in Table 1, with the increase in the confidence level τ, user unit electricity purchase costs tend to decrease, while HCNG purchase costs gradually increase. The expansion of the uncertainty range has a more significant impact on electricity purchase costs, primarily because electricity procurement is jointly influenced by multiple uncertainties, including wind power output, load fluctuations, and combustion reaction characteristics. This results in a relatively large variation in electricity costs, with a maximum difference of up to 6319.6 CNY. However, this variation remains within an acceptable and adjustable cost range. Compared to Case 4, Case 3 (τ = 0.95) effectively mitigates the impact of large uncertainty fluctuations by reducing electricity purchases, thereby improving overall system economic performance. Meanwhile, the increase in HCNG purchase costs under different *τ* values is more gradual. Notably, the cost in Case 1 is close to that in Case 4, and the difference between Cases 2 and 3 is less than 0.246%, indicating minor fluctuations. This demonstrates that the HCNG procurement strategy remains economically efficient while effectively handling multiple uncertainties.

(3) Operation of the HCNGN and Physical Characteristics of HCNG

**Figure 11:**
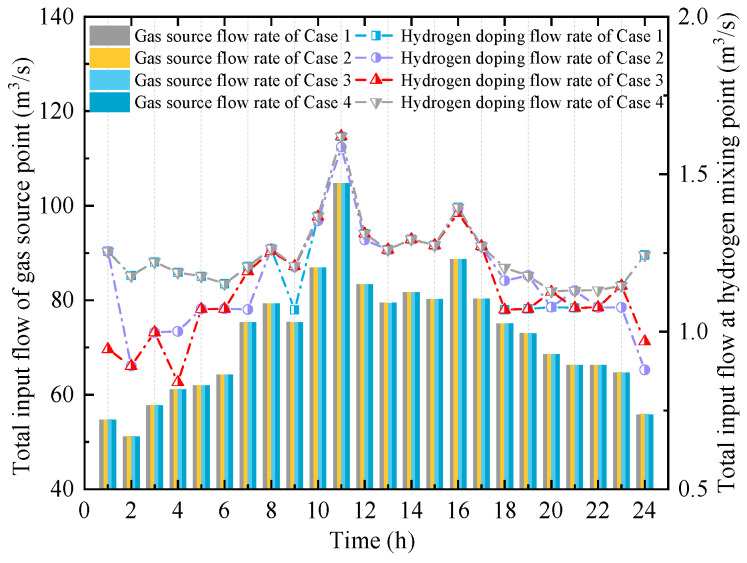
Total input flow of gas sources and hydrogen blending nodes in Cases 1–4.

As the coupling interface between user units and the urban distribution network, the HCNGN is affected by multiple uncertainties, including wind power, load fluctuations, and combustion reaction dynamics. These uncertainties influence the operational strategy of the HCNGN. Therefore, it is essential to analyze its operational behavior and the physical characteristics of HCNG under different confidence levels *τ*. Figure 11 presents the total input flow rates at both the gas source and hydrogen blending points for Cases 1–4. Correspondingly, Table 2 summarizes the natural gas supply costs at the gas source under each scenario.

With the increase in *τ*, the variation in total input flow at the gas source becomes smoother over time, indicating that the impact of multiple uncertainties on gas supply is relatively minor. This is mainly due to the operational flexibility of the HCNGN, which helps buffer the effects of uncertainty fluctuations. To further evaluate the economic performance of natural gas supply, Table 2 shows that when *τ* ranges from 0.85 to 0.95, the gas supply costs in Case 2 and Case 3 differ by no more than 0.007%. This demonstrates that the proposed EHUIES scheduling strategy can effectively and stably control large increases in natural gas supply costs, even under higher levels of uncertainty.

**Figure 12:**
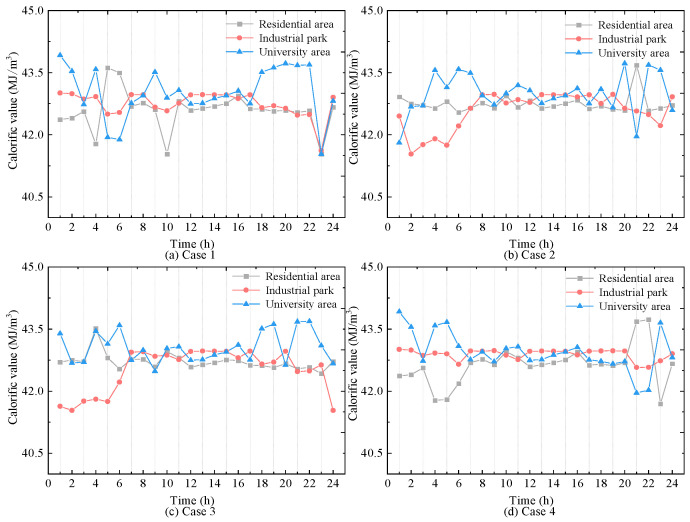
(**a**) Calorific value of HCNG in Case 1; (**b**) calorific value of HCNG in Case 2; (**c**) calorific value of HCNG in Case 3; (**d**) calorific value of HCNG in Case 4.

**Figure 13:**
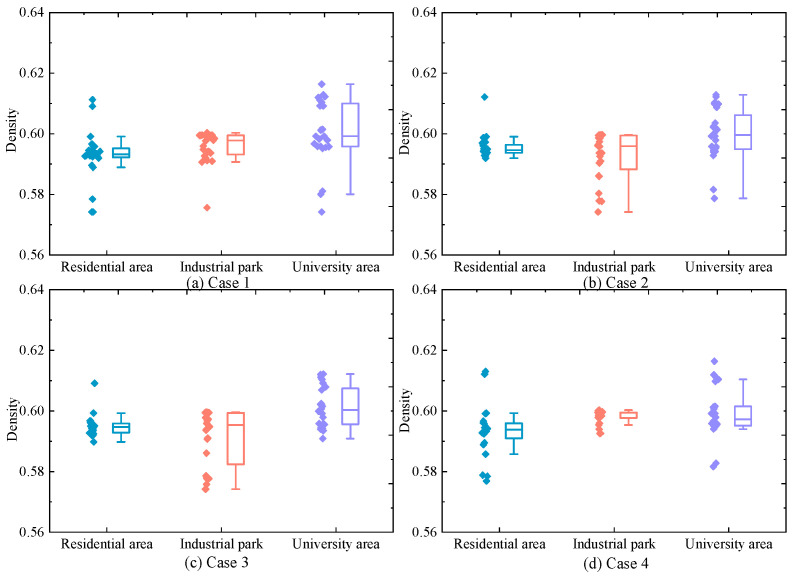
(**a**) Density of HCNG in Case 1; (**b**) density of HCNG in Case 2; (**c**) density of HCNG in Case 3; (**d**) density of HCNG in Case 4.

Furthermore, to analyze the variation in HCNG physical characteristics under different scenarios, the calorific value and density of HCNG for Cases 1–4 are shown in Figure 12 and Figure 13, respectively. It can be observed that although the HCNG calorific value and density obtained through adjusted HCNGN operation strategies differ slightly across different τ values, they remain within a relatively narrow fluctuation range. In particular, during time periods 11–18, the HCNG calorific values across Cases 1–4 exhibit similar trends. As shown in Figure 12, this is mainly due to the similar total input flow rates at the hydrogen blending points during these hours, resulting in relatively stable hydrogen molar fractions.

(4) Actual steady-state combustion reaction margin of HCNG

**Figure 14:**
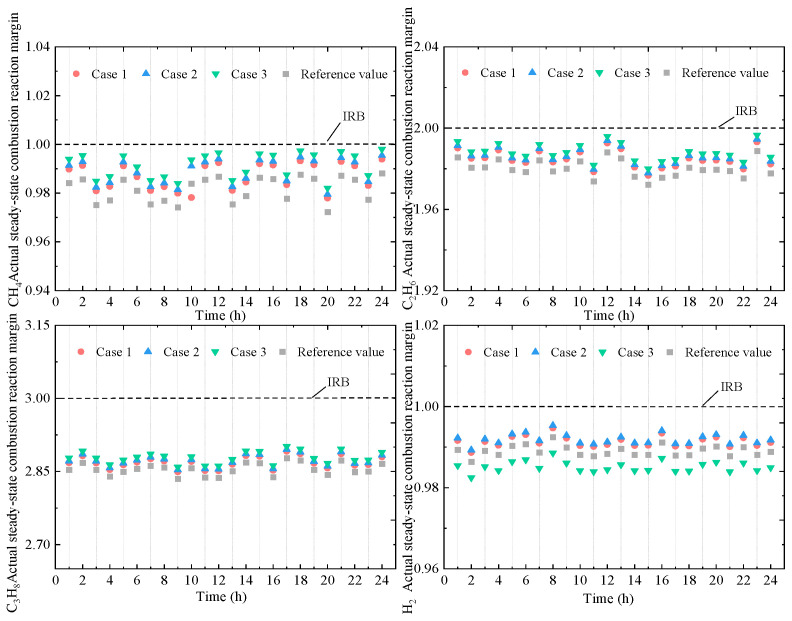
Actual steady-state combustion reaction margin of Cases 1–3.

When uncertainty is present in the HCNG combustion reaction, the actual combustion outcomes of the gas mixture vary across different scenarios, leading to differing impacts on the operational characteristics of the HBGT. Therefore, the steady-state combustion margin under Cases 1–3 is further analyzed, as illustrated in Figure 14.

With variations in the confidence level τ, the actual steady-state combustion margins of various gas components deviate from the ideal reaction boundary (IRB) to meet the power generation requirements of the HBGT. These margins do not remain constant over time. Among the components, methane, ethane, and hydrogen exhibit combustion margins that are generally closer to the IRB. Moreover, the actual steady-state combustion margins tend to approach the upper and lower bounds of the adjusted confidence interval of the reference values. Compared to Cases 1 and 2, Case 3 represents the scenario with the largest fluctuation in combustion margins. Notably, the hydrogen combustion margin in Case 3 is significantly lower than the reference value, indicating a preference under *τ* = 0.95 for relaxing hydrogen combustion conditions to increase the hydrogen blending ratio in HBGT operation. Therefore, in practical applications, it is necessary to improve the combustion conditions of the HBGT to accommodate the operational requirements of the EHUIES under different scenarios.

(5) Analysis of System Optimization Objective Results

To analyze the optimization results under different scenarios, this section focuses on two key aspects: the total system operating cost and the objective deviation. The results for Cases 1–4 are summarized in Table 3.

As shown in Table 3, the objective deviation increases with higher confidence levels, while the total system operating cost shows a slight decrease. Notably, although there is a significant increase in objective deviation between Case 2 and Case 3, the corresponding system operating costs differ by no more than 0.011%. At the same time, the cost trends in Cases 1–3 are generally lower than or comparable to those in Case 4, indicating that the proposed scheduling strategy maintains strong economic performance even under significant multi-source uncertainty. In addition, the operating costs of water electrolysis units, wind curtailment costs, and electricity purchase costs for user units in Cases 1–3 exhibit more notable variations. When *τ* ranges from 0.85 to 0.95, the variation in other related costs remains relatively small, with the smallest difference being only 0.091%. This suggests that uncertainties in the urban distribution network have a more pronounced effect on certain cost components. In summary, as the fluctuation range of multi-source uncertainty increases, the proposed scheduling strategy leverages internal coordination within EHUIES to effectively constrain cost variation within a narrow economic range.

**Figure 15:**
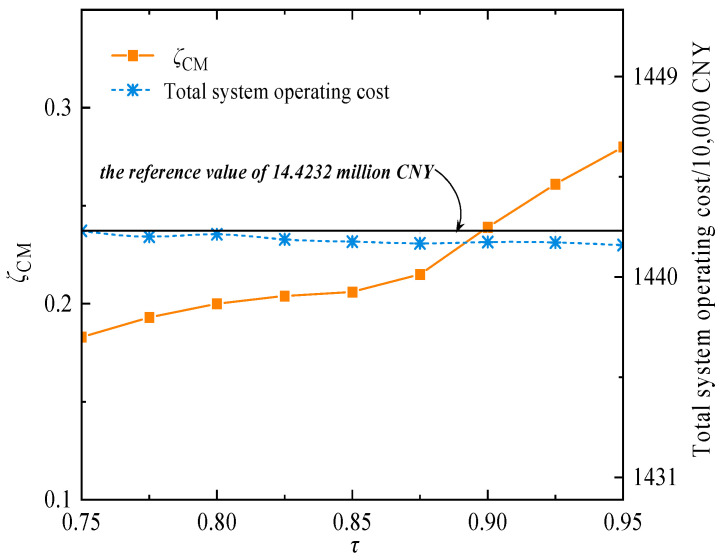
Solution results analysis of system optimization objectives with different confidence levels.

Under the case study settings, for any confidence level *τ* within the range from 0.75 to 0.95 with a step size of 0.025, the trends of total system operating cost and objective deviation with respect to *τ* are further analyzed, as shown in Figure 15. It is evident that as τ increases from 0.75 to 0.95 in increments of 0.025, the objective deviation rises noticeably. However, the total system operating cost remains within a narrow fluctuation range, stabilizing below 14.4232 million CNY. This indicates that the impact of multiple uncertainties on the system exhibits a degree of randomness. Meanwhile, the proposed method maximizes the deviation caused by uncertainties at different time points; however, to ensure that the total expected cost is not exceeded, it formulates reasonable and precise economic dispatch strategies based on available uncertainty information. Consequently, this approach reduces the conservatism of system decision-making.

**Figure 16:**
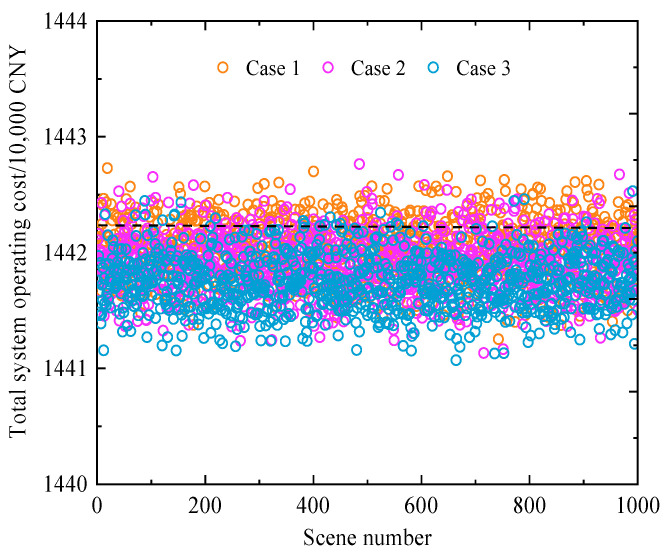
Monte Carlo sampling results of total system operation costs in Cases 1–3.

Furthermore, to further validate the effectiveness of the proposed model and method, three confidence levels *τ* = 0.75, 0.85, and 0.95 were selected. Using the Monte Carlo sampling method, *N_mc_* = 1000 sets of wind power, load, and combustion reaction scenarios were randomly generated for deterministic solving. Since the interaction of maximum objective deviations at different time points causes variations in system costs, it is necessary to consider cases where costs may exceed those obtained from the deterministic model. The Monte Carlo statistical results of the total system operating cost for Cases 1–3 are shown in Figure 16.

As shown in Figure 16, the total system operating costs for Cases 1–3 remain within a relatively narrow fluctuation range. In particular, the cost ranges of Case 2 and Case 3 are closely aligned across multiple scenarios. Multi-scenario analysis within the corresponding fluctuation intervals indicates that the SOI-IGDT approach effectively manages varying levels of uncertainty risk. When *τ* = 0.95, the model ensures that the total cost in most scenarios does not exceed the expected threshold, indicating a low occurrence of “constraint violations” and demonstrating strong economic efficiency and reliability. In contrast, at *τ* = 0.75, the probability of exceeding the expected cost is significantly higher, reflecting a weaker resistance to uncertainty. These results validate the robustness and effectiveness of the proposed method under multi-source uncertainty.

### 4.2. The Impact of Hydrogen Blending Ratio on User Carbon Emissions at Different Confidence Levels

**Figure 17:**
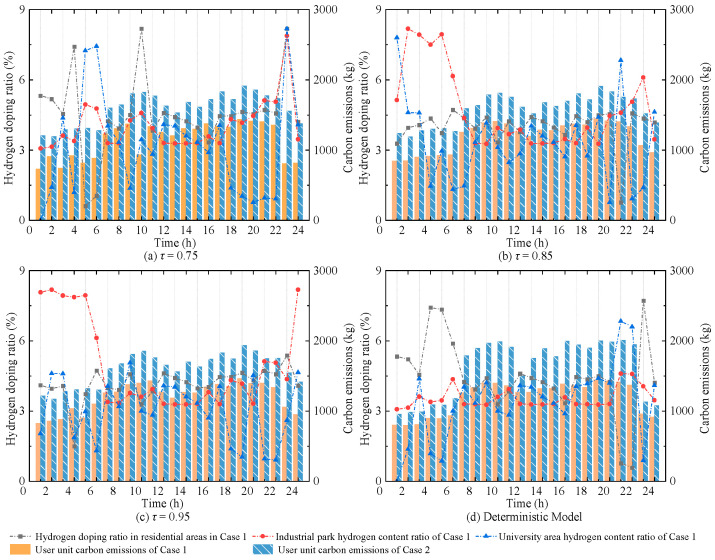
Carbon emissions of user units in Cases 1–2 and the hydrogen blending ratio of Case 1 with different confidence levels.

This section investigates the variations in hydrogen blending ratio and user-level carbon emissions with increasing confidence levels. Two scenarios are compared, using the traditional natural gas-based urban integrated energy system as the reference. Figure 17 shows the user-level carbon emissions for Case 1 and Case 2, as well as the hydrogen blending ratio in Case 1 under different confidence levels.

- Case 1: HCNG is supplied through HCNGN for HBGT-based power generation at urban end-user units.
- Case 2: Natural gas is supplied through the conventional gas network for gas turbine-based power generation at urban end-user units.

As shown in Figure 17, compared to the deterministic model, the hydrogen blending ratio in Case 1 is adaptively adjusted at different time periods with the increase in τ, in response to varying levels of uncertainty. Correspondingly, the carbon emissions exhibit a slight increase. Due to the limited variation in hydrogen input at blending points, the hydrogen blending ratio for user units in Case 1 during 11:00–18:00 remains relatively stable. When τ exceeds 0.85, the associated carbon emissions also fluctuate within a narrow range. In contrast, as observed from Figure 17a–d, the carbon emissions from gas turbines in Case 2 are more significantly affected by multiple uncertainties. The maximum deviation in total carbon emissions in Case 2 reaches up to 1620.86 kg compared to the optimization scheduling results of the urban integrated energy system supplied by traditional natural gas, while the corresponding deviation in Case 1 is only 1171.01 kg. This highlights the effectiveness of hydrogen blending in reducing carbon emissions under multiple uncertainties. By optimally adjusting the hydrogen blending ratio in HCNG, more accurate and reasonable control of user-level carbon emissions can be achieved.

Furthermore, the carbon reduction rate and hydrogen substitution density of user units under different confidence levels are analyzed, as shown in Table 4. It can be observed that at *τ* = 0.75, the carbon reduction rate is close to that of the deterministic model. However, when uncertainty is considered, both the carbon reduction rate and hydrogen substitution density are slightly lower than those of the deterministic case. When *τ* ranges from 0.85 to 0.95, both indicators fluctuate within a narrow range, with a maximum deviation of only 0.49% in carbon reduction rate and 0.07 kg/m^3^ in hydrogen substitution density.

### 4.3. Analysis of System Optimization Results of the Soi-Igdt Model Considering Exergy Enhancement

Based on the SOI-IGDT framework, this section sets up two scenarios to analyze the impact of exergy enhancement on the optimal scheduling of the EHUIES:

- Case 1: The EHUIES optimal scheduling model is constructed using the SOI-IGDT without considering exergy enhancement.
- Case 2: The EHUIES optimal scheduling model is constructed using the SOI-IGDT with exergy enhancement considered.

**Figure 18:**
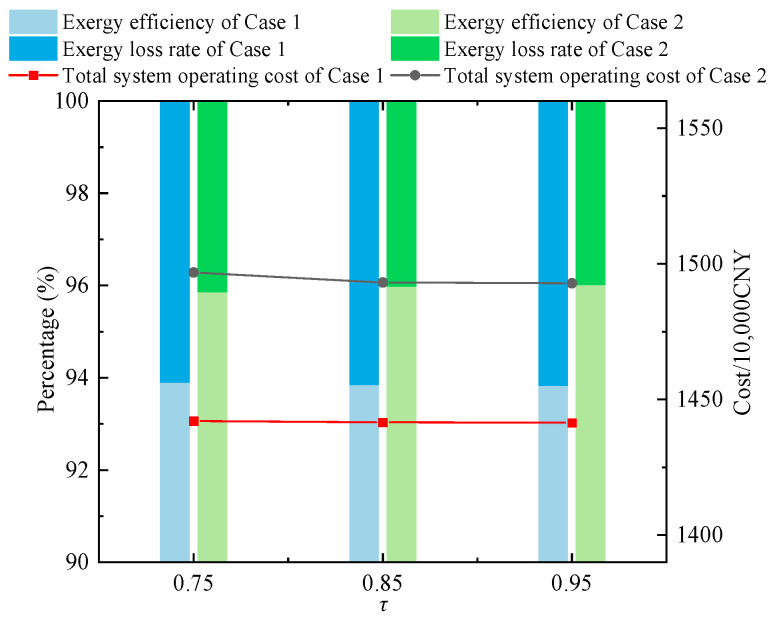
Comparison of total system operation cost and exergy efficiency in Cases 1–2 with different confidence levels.

It is worth noting that the corresponding exergy efficiency and total system operating cost are obtained based on the system optimization results in Section 4.1. Figure 18 presents the total operating costs and exergy efficiencies of Case 1 and Case 2 under different confidence levels. As τ increases, the variation in exergy efficiency in Case 1 is relatively small and consistently lower than that in Case 2, indicating the effectiveness of the method adopted in Case 2 in enhancing exergy performance. Moreover, when τ ranges from 0.85 to 0.95, Case 2 achieves an improvement in exergy efficiency while maintaining the total operating cost within a narrow fluctuation range.

**Figure 19:**
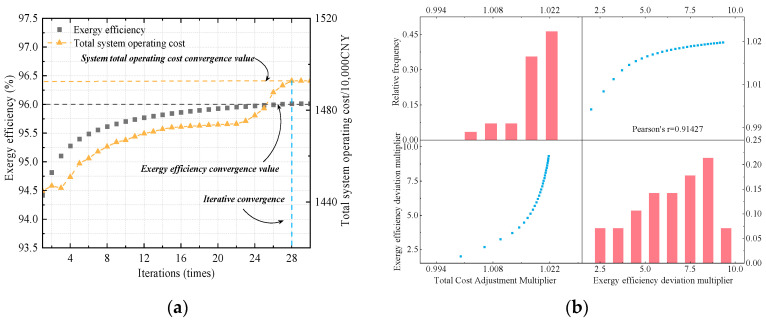
(**a**) Total system operation cost, exergy efficiency iterative convergence curve; (**b**) multiplier correlation analysis when τ = 0.95.

Taking *τ* = 0.95 as an example, the convergence performance of the proposed method in Section 3 is illustrated. The iterative convergence curves of total system operating cost and exergy efficiency are shown in Figure 19a. After 28 iterations, both the exergy efficiency and total cost become nearly stable. The results also indicate that achieving an exergy efficiency above 96.0% requires a significantly higher operating cost, suggesting a trade-off between performance and cost. Moreover, the two multipliers in the model adopt different update strategies. Figure 19b provides a detailed statistical analysis of the correlation and variation trends between the total cost adjustment multiplier and the exergy efficiency deviation multiplier during the iterative process. As shown in Figure 19b, the total cost adjustment multiplier exhibits a relatively small growth after reaching 1.022, while the exergy efficiency deviation multiplier fluctuates more significantly due to the influence of the initial update parameters. Additionally, the Pearson correlation coefficient between the two multipliers reaches 0.914, indicating a strong positive correlation and suggesting that the update directions of the two multipliers are relatively consistent throughout the iterative process.

## 5. Conclusions

This paper employs the SOI-IGDT method, considering exergy enhancement, to investigate the optimal scheduling strategy of EHUIES under multiple uncertainties. First, the stochastic characteristics of multiple uncertainties, including wind power, load, and combustion reactions, are analyzed. A quantitative analysis is conducted specifically for combustion reaction uncertainties, and confidence interval classifications for multiple uncertainties are presented. Second, addressing the limitations of the existing IGDT approach, this study integrates chance constraints with IGDT based on available uncertainty information support. To reduce the conservatism of the original system, the deviation factor setting is removed. For the symmetric consistency issue of the original target deviation, a time-correlated deviation priority selection mechanism is introduced to achieve mean approximation across the entire scheduling horizon, thus expanding the solution space. Consequently, an SOI-IGDT model is proposed to construct an EHUIES optimal scheduling framework under multiple uncertainties. Since the system optimization model with chance constraints is difficult to solve directly, a specific equivalent deterministic transformation method is provided. Additionally, to enhance high-quality energy utilization in EHUIES, total cost adjustment and exergy efficiency deviation multipliers are introduced within SOI-IGDT to enable iterative optimization, thereby achieving improved exergy efficiency. Taking a practical 186-node distribution network system in a certain city, coupled with a 20-node HCNGN as the test case, the following conclusions are drawn from the simulation analysis:

- With the increase in confidence level, the SOI-IGDT-based optimal scheduling strategy for EHUIES can effectively handle multiple uncertainties while still satisfying the system’s energy supply demands. When τ ranges from 0.85 to 0.95, the optimization results tend to stabilize. In addition, the hydrogen blending ratio is flexibly adjusted in response to changes in τ, maintaining the carbon reduction rate and hydrogen substitution density within narrow variation ranges, with a maximum deviation of only 0.49% and 0.07 kg/m^3^, respectively.
- Based on the available uncertainty information, the target deviation obtained through SOI-IGDT varies across different time periods. This allows for the development of adaptive EHUIES scheduling strategies tailored to specific operational scenarios under multiple uncertainties, thereby enhancing the model’s practical applicability.
- Compared with the case without exergy enhancement, there exists a trade-off relationship between exergy efficiency and total system operating cost. A significant improvement in exergy efficiency results in a corresponding increase in operating cost. For instance, when τ = 0.95, the actual exergy efficiency improves by 2.18%, while the total operating cost increases by 3.44%. Furthermore, the Pearson correlation coefficient between the two multipliers reaches 0.914, indicating a strong positive correlation and validating the effectiveness of the proposed SOI-IGDT model considering exergy enhancement.

## Figures and Tables

**Table 1:** Electricity and HCNG purchase cost of user units in Cases 1–4.

Case	1	2	3	4
User unit electricity purchase cost/CNY	11,385.46	6328.38	5065.86	6074.84
User unit purchase HCNG cost/CNY	49,507.63	51,315.03	51,441.39	50,547.48

**Table 2:** Cost of gas source supply in Cases 1–4.

Case	1	2	3	4
Natural gas supply cost/10,000 CNY	1418.37	1418.82	1418.92	1418.36

**Table 3:** Solution results analysis of system optimization objectives in Cases 1–4.

Case	1	2	3	4
Classification mean deviation	0.183	0.206	0.280	/
Operation cost of water electrolysis device/CNY	130,547.72	125,499.35	123,936.62	132,950.64
Operation cost of thermal power unit/CNY	24,542.41	23,641.93	23,719.10	28,062.34
Cost of HCNG purchased by user unit/CNY	49,507.63	51,315.03	51,441.39	50,547.48
Cost of electricity purchased by user unit/CNY	11,385.46	6328.38	5065.86	6074.84
Carbon emission cost/CNY	5938.63	6224.25	6259.22	6190.92
Equipment operation and maintenance cost/CNY	15,038.89	14,634.38	14,647.68	16,015.33
Gas supply cost of natural gas source/10,000 CNY	1418.37	1418.82	1418.92	1418.36
Total system operation cost/10,000 CNY	1442.07	1441.59	1441.43	1442.32

**Table 4:** Carbon reduction rate and hydrogen substitution density of user units with different confidence levels.

Scenario Classification	τ			Deterministic Model
	0.75	0.85	0.95	
Carbon reduction rate/%	25.96	24.11	24.60	26.03
Hydrogen substitution density/kg·m^−3^	13.10	11.76	11.83	14.08

## Data Availability

Data available upon request due to restrictions (e.g., privacy, legal, or ethical reasons). The data presented in this study are available upon request from the corresponding author. The data are not publicly available due to the restrictions, which include the fact that the data obtained from the City Power Authority currently do not have the authorization for public release.
